# Higher adherence to (poly)phenol-rich diet is associated with lower CVD risk in the TwinsUK cohort

**DOI:** 10.1186/s12916-025-04481-5

**Published:** 2025-11-27

**Authors:** Yong Li, Xinyu Yan, Yifan Xu, Robert Pope, Tim D Spector, Mario Falchi, Claire J Steves, Jordana T Bell, Kerrin S Small, Cristina Menni, Rachel Gibson, Ana Rodriguez-Mateos

**Affiliations:** 1 Department of Nutritional Sciences, School of Life Course and Population Sciences, Faculty of Life Sciences and Medicine, King’s College London, London, UK; 2 Department of Twin Research and Genetic Epidemiology, School of Life Course and Population Sciences, Faculty of Life Sciences and Medicine, King’s College London, London, UK; 3 Department of Pathophysiology and Transplantation, Università Degli Studi Di Milano, Milan, Italy

**Keywords:** (Poly)phenol intake, (Poly)phenol-rich dietary score, Urinary metabolites, Metabolic signature, Cardiovascular risk scores

## Abstract

**Background:**

Previous studies have reported inverse associations between total (poly)phenol intake or specific subclasses of (poly)phenols, estimated from dietary questionnaires, and cardiovascular disease (CVD) risk. However, no studies have examined (poly)phenol-rich dietary patterns and their corresponding urinary metabolite profiles in relation to CVD risk. This study investigated the associations between a (poly)phenol-rich dietary score (PPS-D), its urinary metabolic signature (PPS-M), and longitudinal CVD risk in the TwinsUK cohort.

**Methods:**

We included 3110 participants (followed up for 11.20 ± 7.03 years) from TwinsUK who completed the EPIC-Norfolk Food Frequency Questionnaire with longitudinal data. A subset of 200 participants provided spot urine samples, in which 114 (poly)phenol metabolites were quantified using ultra-high-performance liquid chromatography-mass spectrometry (UHPLC-MS) to objectively measure (poly)phenol exposure. Associations between the PPS-D or PPS-M and CVD risk scores (ASCVD risk score and HeartScore), and biomarkers of CVD risk (blood pressure and lipid profile) were assessed using linear mixed models, adjusting for covariates and multiple testing (FDR < 0.05).

**Results:**

PPS-D was negatively associated with ASCVD risk score (stdBeta: − 0.05 (− 0.07, − 0.04)) and Heartscore (stdBeta: − 0.03 (− 0.04, − 0.01)) (FDR-adjusted *p* < 0.01) in the overall population (*n* = 3,110). In the subgroup with urinary metabolites (*n* = 200), such significant associations were partially replicated through metabolites of flavonoids, phenolic acids, and tyrosols that significantly negatively associated with the ASCVD risk score, HeartScore, diastolic blood pressure (DBP), and positively with high-density lipoprotein cholesterol (HDL-C). In addition, a higher PPS-M was correlated with elevated HDL-C and lower blood pressure, ASCVD risk score, and HeartScore.

**Conclusions:**

Higher adherence to a (poly)phenol-rich diet is associated with lower CVD risk, with consistent associations observed through urinary metabolite profiles, highlighting the long-term cardiovascular  benefits of (poly)phenol consumption, particularly flavonoids and phenolic acids.

**Supplementary Information:**

The online version contains supplementary material available at 10.1186/s12916-025-04481-5.

## Background

(Poly)phenols are a diverse group of plant-derived secondary metabolites found in a variety of plant-based foods and beverages, including tea, coffee, fruits, vegetables, nuts, seeds, cocoa and soy products [1, 2]. Growing evidence suggests that (poly)phenols play a role in reducing the risk of cardiovascular diseases (CVD) [3]. Indeed, (poly)phenols have been shown to modulate the nitric oxide pathway, which plays a crucial role in maintaining vascular homeostasis [4]. Additional mechanisms, such as inhibiting platelet aggregation, reducing inflammation, preventing low-density lipoprotein oxidation, and improving blood lipid profiles, further support their beneficial effects on cardiovascular health [5, 6]. Numerous randomised controlled trials have also demonstrated that (poly)phenol consumption increases flow-mediated dilation, reduces blood pressure, and lowers CVD mortality [7–9].

However, a major challenge lies in accurately assessing habitual (poly)phenol intake in free-living populations. Most dietary assessment tools used in large cohort studies [10–12] have not been validated for estimating (poly)phenol intake [13]. Furthermore, comprehensive databases like Phenol-Explorer [14] and the USDA databases [15–17] still lack detailed information on the (poly)phenol content of many foods, and factors like species, storage, and processing methods are often overlooked. The accurate measurement of the (poly)phenol content of foods requires advanced analytical techniques and authentic chemical standards, which are not widely available [13]. Due to these limitations in both dietary assessment and analytical methods, the long-term impact of (poly)phenol intake on cardiovascular health outcomes remains poorly understood.

To address these limitations, our research group developed a novel (poly)phenol-rich diet score (PPS) [18] designed to characterise (poly)phenol-rich diets, based on the intake of (poly)phenol-rich foods included in the widely used EPIC (European Prospective Investigation into Diet and Cancer)-Norfolk food frequency questionnaire (FFQ) [19]. This score provides a reliable and accessible method for evaluating an individual’s adherence to a (poly)phenol-rich diet in a free-living population [18]. In addition, metabolomics has emerged as a powerful tool in nutrition research, enabling the identification of biomarkers that reflect adherence to specific dietary patterns [20–23]. Building on the PPS framework, we developed a metabolic signature to objectively assess a (poly)phenol-rich diet, using a high-throughput, targeted metabolomics approach that quantifies a panel of 51 (poly)phenol metabolites [24].

**Fig. 1:**
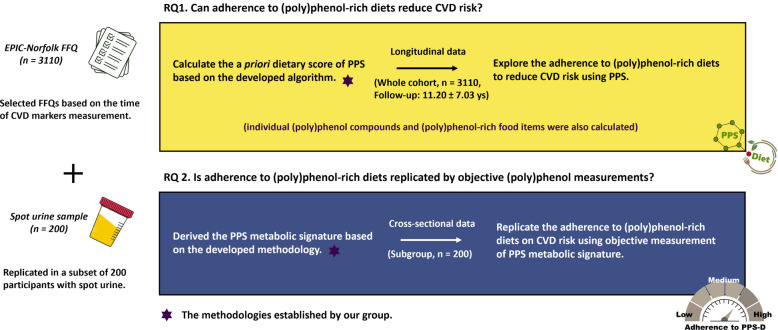
Schematic representation of the study design with longitudinal and cross-sectional data. The key research questions, data, and analytical workflow to address the questions are outlined

By linking cardiovascular risks to (poly)phenol intake through the PPS and validating these associations with the PPS metabolic signature and urinary (poly)phenol metabolites in large, population-based cohorts, we aim to clarify the long-term effect of (poly)phenol consumption. In this study, we analysed a cohort of 3110 twins from the TwinsUK registry to assess the impact of adherence to (poly)phenol-rich diets on CVD risk, as quantified by the PPS derived from the EPIC-Norfolk FFQ (PPS-D). To further validate our findings, we analysed urinary (poly)phenol metabolites and the PPS metabolic signature (PPS-M) using spot urine samples collected from a subset of 200 participants (Fig. 1).

## Methods

### Study population

This study included 3110 participants (monozygotic twin pairs (MZ) = 760, dizygotic twin pairs (DZ) = 790, 5 pairs of related twins for whom zygosity information was not available) enrolled from the TwinsUK registry, a national database of adult twins recruited voluntarily without specific screening criteria [25, 26]. Study visits were conducted on average of 11.20 ± 7.03 years apart between the baseline (from July 1992 to February 2020) and follow-up (from April 1997 to May 2023) time points (3,110 participants) with cardiovascular measurements and food frequency questionnaires (FFQs) collected at each time point.

To replicate the FFQ-derived findings, random spot urine samples collected from 200 participants were analysed for urinary (poly)phenol metabolites. Urine collections were conducted from November 2012 to December 2014, with an average of 2.33 ± 3.68 years apart from the CVD risk assessment for each participant. Ethical approval for the TwinsUK cohort was obtained from St Thomas Hospital Research Ethics Committees, and all participants provided informed written consent (REC ref EC04/015).

### Dietary assessment tools for the estimation of (poly)phenol intake

Participants completed the EPIC-Norfolk FFQ [19] (available *n* = 3,110) [27]. There are four versions of EPIC-Norfolk FFQs, developed for UK adults, that have been used in the study. For FFQ version 1, the EPIC-Norfolk FFQ collects the dietary intake of 130 food items that are consumed in the UK diet that designed and validated to estimate key nutrients and energy intake in the past year [19, 28], whereas for FFQ version 2–4, the EPIC-Norfolk FFQ is an adapted 131-item questionnaire validated against pre-established nutrient biomarkers for the EPIC Norfolk [29]. All the FFQ data were processed by the same trained researcher according to the same protocol to streamline the processing of the FFQ records as well as to re-shape the data into a format that is relevant for dietary index calculations.

The questionnaire offers 9 frequency options ranging from ‘Never or less than once a month’ to ‘more than 6 times per day’. A separate section of the questionnaire also collected information on the types of milk, cereals, cooking fats, and the amount of visible fat consumed in meals. To minimise the impact of outliers in dietary intake, participants were excluded based on the following criteria: (i) more than 10 missing ticks; (ii) energy intake outside the range of 500–3500 kcal/day for women and 800–4000 kcal/day for men; (iii) an energy intake to BMR ratio falling outside the mean ± 2SD (0.48–2.18 for this dataset) within the study population.

### Dietary (poly)phenol intake estimation with FFQs

Estimating (poly)phenol intake for each food item listed in the EPIC-Norfolk FFQs was accomplished through a home database established with data from the online open-access Phenol-Explorer database [30], the USDA database and several published studies [31–53]. Data derived from normal phase High-Performance Liquid Chromatography (HPLC), chromatography and chromatography after hydrolysis methods were utilised. (Poly)phenol content data of compounds with sugar moieties were converted into the corresponding amount of aglycones to be summarised with data from other sources. Procyanidin data obtained from normal phase HPLC were prioritised, with chromatography data being used when HPLC data were unavailable. For cooked foods, if only the raw data food source was available, the processed yield factor from the Phenol-Explorer database multiplied by the unprocessed raw food content was applied to determine the (poly)phenol content of cooked processed foods. If no yield factor was available, a factor of a similar food item or similar processing method of the same item was applied instead. Daily (poly)phenol intake (mg/day) was calculated by multiplying food intake (g/day) by the corresponding (poly)phenol content from the database (mg/100 g) and dividing by 100. Total and subclasses of (poly)phenols, followed by the classification of Phenol-Explorer, were calculated by summing up all compounds within the group.

### Development of a (poly)phenol-rich dietary score and its metabolic signature

A (poly)phenol-rich dietary score, PPS, was developed to assess adherence to a diet rich in (poly)phenols [18]. The PPS was based on the relative intake of 20 (poly)phenol-rich foods in the EPIC-Norfolk FFQ, including tea, coffee, red wine, wholegrains, breakfast cereals, chocolate and cocoa products, berries, apples and apple juice, pears, grapes, plums, citrus fruits and citrus juice, potatoes and carrots, onions, peppers, garlic, green vegetables, pulses, soybeans, nuts, and olive oil. Participants were scored by the quintiles of their intake of each food group in the study population. The subjects in the highest quintile scored 5, and those in the lowest quintile scored 1. The PPS was calculated as the total score of all the 20 food group scores ranging from 20 to 100.

Metabolic signatures represent a combination of metabolites that evaluate adherence to a specific diet to characterise dietary patterns [20, 21]. The metabolic signature of PPS was established as an objective tool to assess (poly)phenol intake and reflects adherence to (poly)phenol-rich diets [24]. The signature was derived from 51 metabolites, with individual penalised weights for each metabolite determined through ridge regression, as described in prior research [24].

Here, the acronyms PPS-D and PPS-M are used to signify the PPS dietary score and PPS metabolic signature.

### Measurements of CVD risk

In this study, the following parameters were assessed: systolic and diastolic blood pressure (SBP and DBP, mmHg), total cholesterol (TC, mmol/l), high-density lipoproteins cholesterol (HDL-C, mmol/L), atherosclerotic cardiovascular disease (ASCVD) risk score and HeartScore. Detailed descriptions of these measurements have been previously reported [54–57]. Briefly, SBP and DBP were taken by a trained nurse using an automated cuff sphygmomanometer (OMRON HEM713C) after the participant had been seated for at least three minutes, with three readings recorded. The average of the three readings were used. Blood for TC and HDL-C analysis were taken following a 10-h overnight fast.

The individual ASCVD risk score was calculated using the 10-year ASCVD risk estimator, incorporating factors including sex, ethnicity, age, smoking status, TC, BP, and diabetes history. The score, available online (http://tools.acc.org/ASCVD-Risk-Estimator-Plus/), is categorised into four levels: low risk, borderline risk, intermediate risk, and high risk (< 5.0%; 5.0–7.4%; 7.5–19.9%, and ≥ 20.0%) [58]. This score estimates the 10-year risk of developing hard ASCVD events, such as coronary heart disease death, nonfatal myocardial infarction, and fatal or nonfatal stroke [59]. The HeartScore estimator (https://www.heartscore.org/en_GB/) was also used to assess the 10-year risk of fatal and nonfatal cardiovascular disease events, using sex, age, SBP, TC, HDL-C and smoking status as variables. One of four European risk regions (low, moderate, high, and very high-risk regions) was selected based on standardised CVD mortality rates, with the UK classified into the low-risk region [60, 61]. The Systematic COronary Risk Evaluation (SCORE) model as a well-designed algorithm was implemented in the HeartScore estimator. There are two models in the latest SCORE versions, including SCORE2 [60] and SCORE2-OP [61] that estimate 10-year fatal and nonfatal CVD risk in Europeans aged 40–69 years (SCORE2) and those aged over 70 (SCORE2-OP) who have no prior CVD or diabetes [60, 61].

### Assessment of covariates

The analysis was adjusted for several covariates: age, sex, ethnicity, body mass index (BMI, kg/m^2^), daily energy intake (kcal/day), alcohol consumption (g/day), sodium intake (mg/day), and fibre intake (g/day). Age and relatedness between samples were obtained from a self-reported lifestyle questionnaire [25]. Participants’ weight and height were measured by trained nurses during clinical visits to calculate BMI (kg/m^2^) according to standardised protocols [25]. Energy intake, a common covariate in nutritional epidemiology [62], is included since individuals with higher energy intake typically consume more food/nutrients, including (poly)phenol-rich sources, whereas excessive energy intake may promote obesity and metabolic disorders. Alcoholic beverages, in particular, wine, is a significant source of (poly)phenols, e.g. anthocyanins and flavan-3-ols [63], and it represents a well-established factor related to CVD [64]. High sodium intake is a leading dietary cause of hypertension [65], whereas diets high in sodium, such as processed foods, might be low in (poly)phenol-rich whole foods, e.g. fruits and vegetables. In addition, (poly)phenol-rich foods, especially fruits, vegetables and wholegrains, are primary sources of fibre, whereas promoting higher fibre intake may benefit CVD and hypertension patients [66]. As a result, daily intakes of energy [58], alcohol [58], sodium and fibre [67, 68] were chosen as covariates and calculated from FFQs using the FFQ EPIC and Nutrition Tool for Analysis (FETA) software [69].

### Analysis of (poly)phenol metabolites in urine samples using UHPLC-MS

In a subset of 200 participants, spot urine samples were collected at their visit time. Samples were processed and analysed using a validated method involving micro-elution solid phase extraction combined with ultra-high-performance liquid chromatography–triple quadrupole mass spectrometry (UHPLC-Q-q-Q MS). Urine collections were conducted from 2012 to 2014 with an average of 2.33 ± 3.68 years apart from the CVD assessment. A total of 114 (poly)phenol metabolites were identified and quantified with authentic standards, as detailed in previous studies [67, 70]. Raw data analysis and calculation were conducted using LabSolutions software (SHIMADZU, Kyoto, Japan). Urinary creatinine levels were measured by Affinity Biomarker Labs (London, UK) using the Jaffe method, and metabolite concentrations (nM) were normalised by creatinine levels (mg/L) and expressed as mmol/g creatinine. The formula to calculate the sample size of the spot urine sample in the subgroup cross-sectional analysis is as follows:

$$\text{sample size}=\frac{{z}_{(1-\frac{\alpha }{2})}^{2}\times p(1-p)}{{d}^{2}}$$

${z}_{(1-\frac{\alpha }{2})}$ is the *Z* value derived from the standard normal distribution, and a 95% confidence level is used here with 1.96 for *Z*-score.

P represents the Expected Prevalence of cardiovascular risk in the UK. According to the British Heart Foundation, the prevalence of coronary heart disease is 3.13% in the Heart & Circulatory Disease Statistics 2023 (https://www.bhf.org.uk/what-we-do/our-research/heart-statistics/heart-statistics-publications/cardiovascular-disease-statistics-2023).

d represents the Precision (margin of error) and is set as 0.03.

As a result, the final sample size required is 129, and we collected 200 urine samples in this study.

### Statistical analysis

Statistical analysis was performed using R version 3.6.2 [71]. Data distribution was assessed graphically with a histogram and Q-Q plot, and log transformation was applied to normalise variables as needed for the analysis. Multiple testing adjustments were made using the Benjamini and Hochberg False Discovery Rate (FDR < 0.05) [72].

The baseline and follow-up time points were used in the whole cohort (3110 participants, followed up for 11.20 ± 7.03 years) to assess the longitudinal changes in cardiovascular health using the linear mixed model (‘lme4’ package in R). The baseline PPS-D/dietary (poly)phenol intake and the change in PPS-D/dietary (poly)phenol intake between two time points measured from FFQ were used as explanatory variables, and cardiovascular risks and markers were used as response variables. Relatedness between samples was included as a random effect, and the models were further adjusted for age, sex, ethnicity, energy intake, alcohol consumption, fibre intake, sodium intake, and BMI. To explore the change in (poly)phenol from baseline to follow-up time point, the baseline age and time duration were used as explanatory variables, and PPS-D/dietary (poly)phenol intake was used as response variable with energy intake adjusted. To explore the change in cardiovascular health markers from baseline to follow-up time point, the baseline age and time duration were also used as explanatory variables, with sex, ethnicity, energy intake, alcohol consumption, fibre intake, sodium intake, and BMI adjusted.

To examine the relationship between urinary (poly)phenol metabolites (explanatory variables) and PPS-D/PPS-M or cardiovascular markers (response variables), linear mixed models were also employed with family-relatedness as a random effect. These models were further adjusted for potential covariates, including age, energy intake, alcohol consumption, fibre intake, sodium intake and BMI.

## Results

### Study population

The demographic characteristics of the cohort (*n* = 3,110) from the TwinsUK registry are shown in Table 1. The majority of the subjects were female (96.7%) and of white ethnicity (99.0%), with an average age and BMI of 51.8 (SD 11.1) years, 25.4 (SD 4.7) kg/m^2^, and 64.4 (SD 11.0) years, 26.2 (SD 4.9) kg/m^2^ in the baseline and follow-up, with an average of 11.20 ± 7.03 years apart, respectively. On average, their ASCVD risk score was at low (0.03 (SD 0.04)) and intermediate risk (0.10 (SD 0.12)) in the baseline and follow-up time, respectively.

**Table 1:** Demographic and cardiovascular characteristics of the study population (*n* = 3,100)

Characteristics	Baseline	Follow-up
Female (*n*/%)	3006 (96.7%)	3006 (96.7%)
MZ (*n*/%)	760 (48.9%)	760 (48.9%)
DZ (*n*/%)	790 (50.8%)	790 (50.8%)
Unknown/other zygosity (*n*/%)	5 (0.3%)	5 (0.3%)
White (*n*/%)	3078 (99.0%)	3078 (99.0%)
Age, years (mean/SD)	51.8 (11.1)	64.4 (11.0)
BMI, kg/m^2^ (mean/SD)	25.4 (4.7)	26.2 (4.9)
Fibre intake, g/day (mean/SD)	17.5 (6.3)	17.6 (6.4)
Energy intake, kcal (mean/SD)	1785.8 (471.9)	1671.6 (459.2)
Alcohol intake, g/day (mean/SD)	8.8 (11.6)	8.3 (12.2)
Sodium intake, mg/day (mean/SD)	2577.9 (815.3)	2532.8 (821.6)
***Cardiovascular risk markers***		
SBP (mmHg) (mean/SD)	123.69 (17.17)	130.30 (17.91)
DBP (mmHg) (mean/SD)	76.94 (10.56)	76.29 (10.61)
TC (mmol/L) (mean/SD)	5.58 (1.16)	5.47 (1.09)
HDL-C (mmol/L) (mean/SD)	1.67 (0.46)	1.88 (0.50)
ASCVD risk score (mean/SD)	0.03 (0.04)	0.10 (0.12)
HeartScore (mean/SD)	2.43 (2.22)	5.4 (4.08)

*MZ *monozygotic, *DZ *dizygotic, *BMI *body mass index, *SBP *systolic blood pressure, *DBP *diastolic blood pressure, *TC *total cholesterol, *HDL-C *high-density lipoproteins cholesterol, *ASCVD risk score *atherosclerotic cardiovascular disease risk score

### Longitudinal changes in (poly)phenol-rich diet score (PPS-D) and estimated (poly)phenol intake

**Fig. 2:**
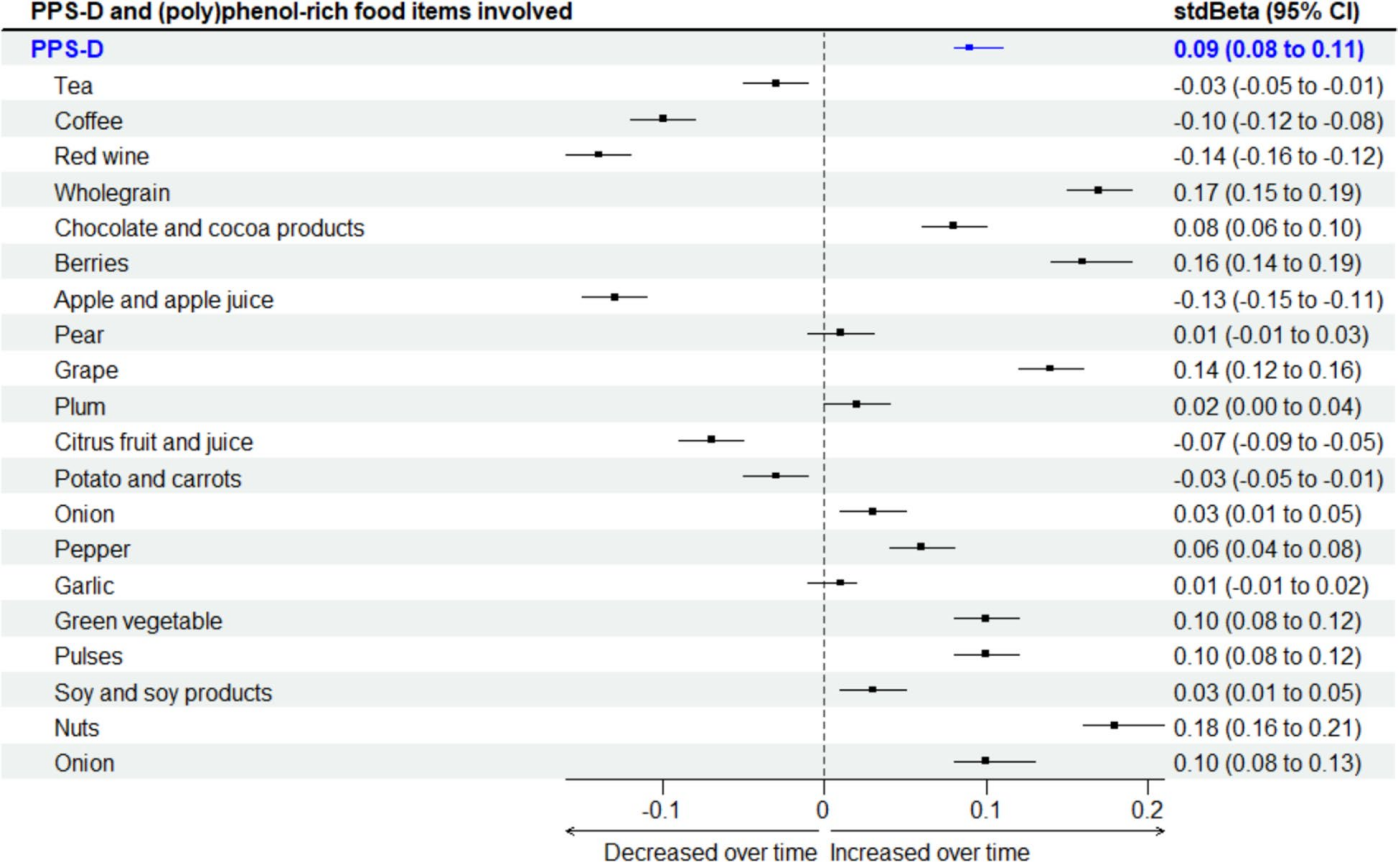
Changes in PPS-D and food consumption from baseline to follow-up time adjusted for relatedness, baseline age of FFQ, and energy intake in the TwinsUK population (*n* = 3,110). PPS-D, (poly)phenol-rich dietary score

The PPS-D of the study population estimated by FFQs was 53.7 (SD 10.4) and 54.3 (SD 10.4) at each time point (PPS dietary score ranging from 20 to 100, the actual PPS dietary score in this study ranging from 25 to 87, Additional file 2: Table S1) and significantly increased from baseline to follow-up (stdBeta: 0.09 (95% CI: 0.08, 0.11), FDR-adjusted *p* < 0.01, Fig. 2). Of all (poly)phenol-rich food items included in the PPS-D, tea and coffee contributed 39.3%, 44.0% at baseline, and 40.1%, and 40.9% at follow-up of the total (poly)phenols, respectively (Additional file 2: Table S1). The average coffee intake was 2.0 ± 1.9 cups per day (380.3 ± 361.5 g day^−1^) to 1.7 ± 1.7 cups per day (319.6 ± 316.1 g day^−1^) from baseline to follow-up, whereas the average tea intake was 2.9 ± 2.0 cups per day (546.3 ± 375.0 g day^−1^) to 2.7 ± 1.9 cups per day (518.7 ± 363.5 g day^−1^) from baseline to follow-up (standardised as 190 g per cup for both coffee and tea according to default portion size in the EPIC-Norfolk FFQ), and both significantly decreased from baseline to follow-up with standard beta of − 0.03 (95% CI: − 0.05, − 0.01) and − 0.10 (95% CI: − 0.12, − 0.08), respectively (all FDR-adjusted *p* < 0.01) (Fig. 2).

(Poly)phenol intake classes and subclasses estimated from FFQs and their food source in baseline and follow-up are shown in Additional file 2: Table S2 and S3. The average total (poly)phenol intake measured from FFQs was 2364.3 (SD 996.3) and 2136.2 (SD 911.3) mg/day at each time point, respectively (Additional file 2: Table S2 and S3). Phenolic acids were the major class of (poly)phenols consumed by our population (contributing 52.5% and 50.4% to total (poly)phenol intake measured from FFQs at each time) (Additional file 2: Table S2 and S3). Total (poly)phenol intake and total phenolic acid intake significantly decreased from baseline to follow-up (stdBeta: −0.11 (95% CI: − 0.13, − 0.09), FDR-adjusted *p* < 0.01; stdBeta: − 0.10 (95% CI: − 0.11, − 0.08), FDR-adjusted *p* < 0.01, respectively Additional file 1: Fig. S1).

As for food sources of (poly)phenols at baseline and follow-up (Additional file 2: Table S2 and S3), non-alcoholic beverages were the main food sources of total (poly)phenols (83.3%), followed by fruits (6.1%) (apples and apple juice 4.1%, orange and orange juice 1.6%, grapes 0.4%), and tomatoes (0.8%), potatoes (0.6%) and red wine (0.6%) at baseline, and non-alcoholic beverages (81.0%), followed by fruits (4.7%) (apple 3.1%, orange 0.9%, and grape 0.7%), red wine (1.2%), tomatoes (1.0%), chocolate (0.9%) and potatoes (0.6%) at follow-up. As for individual foods, coffee contributed the most to total (poly)phenol intake (44.0% baseline and 40.9% follow-up), followed by tea (39.3% baseline and 40.1% follow-up).

To test whether PPS-D have effectively captured habitual (poly)phenol intake, the associations between PPS-D and total and subgroups of (poly)phenol intake were explored. The correlation between PPS-D and FFQs estimated (poly)phenol intake is shown in Additional file 1: Fig. S2. PPS-D was significantly associated with total and 54 out of 57 subgroups of (poly)phenol intake (all FDR-adjusted *p* value < 0.05), with only hydroxycoumarins, alkylmethoxyphenols, and benzoic acids showing no significant association (all FDR-adjusted *p* value > 0.05). Positive associations were found in all significantly associated (poly)phenols, with standard beta ranging from 0.02 (95% CI: 0.001, 0.05 for theaflavins and thearubigins) to 0.36 (95% CI: 0.34, 0.38 for quercetin) (all FDR-adjusted *p* value < 0.05), except for delphinidin (stdBeta: − 0.03, 95% CI: − 0.05, − 0.01) and vanillin (stdBeta: − 0.05, 95% CI: − 0.07, − 0.03), which presented negative associations (all FDR-adjusted *p* value < 0.01).

### Higher adherence to (poly)phenol-rich diets is associated with lower CVD risk

The change in cardiovascular health markers and outcomes is shown in Additional file 1: Fig. S3 (a). CVD risk markers were significantly increased from baseline to follow-up with stdBeta of 0.61 (95% CI: 0.60, 0.62) and 0.58 (95% CI: 0.57, 0.59) for ASCVD risk score and HeartScore, respectively (all FDR-adjusted *p* < 0.01). DBP (stdBeta: − 0.06, 95% CI: − 0.08, − 0.04) and TC (stdBeta: − 0.04, 95% CI: − 0.07, − 0.02) were significantly decreased, whereas SBP (stdBeta: 0.29, 95% CI: 0.28, 0.31) and HDL-C (stdBeta: 0.23, 95% CI: 0.21, 0.25) were significantly increased (all FDR-adjusted *p* < 0.01).

Additional file 1: Fig. S3 (b) and (c) illustrate the dose–response relationships between PPS-D/total (poly)phenol intake and cardiovascular health outcomes (ASCVD risk score and HeartScore), using unadjusted linear regression. A significant inverse association was observed across all analyses, indicating that higher PPS and total (poly)phenol intake were associated with lower cardiovascular risk, as reflected in the ASCVD risk score and HeartScore (stdBeta (95%CI): (poly)phenol and ASCVD/HeartScore: −0.06 (−0.03, −0.08); PPS and ASCVD: −0.04 (−0.01, −0.07); PPS and HeartScore: −0.04 (−0.01, −0.06), all FDR-adjusted *p* < 0.01).

**Fig. 3:**
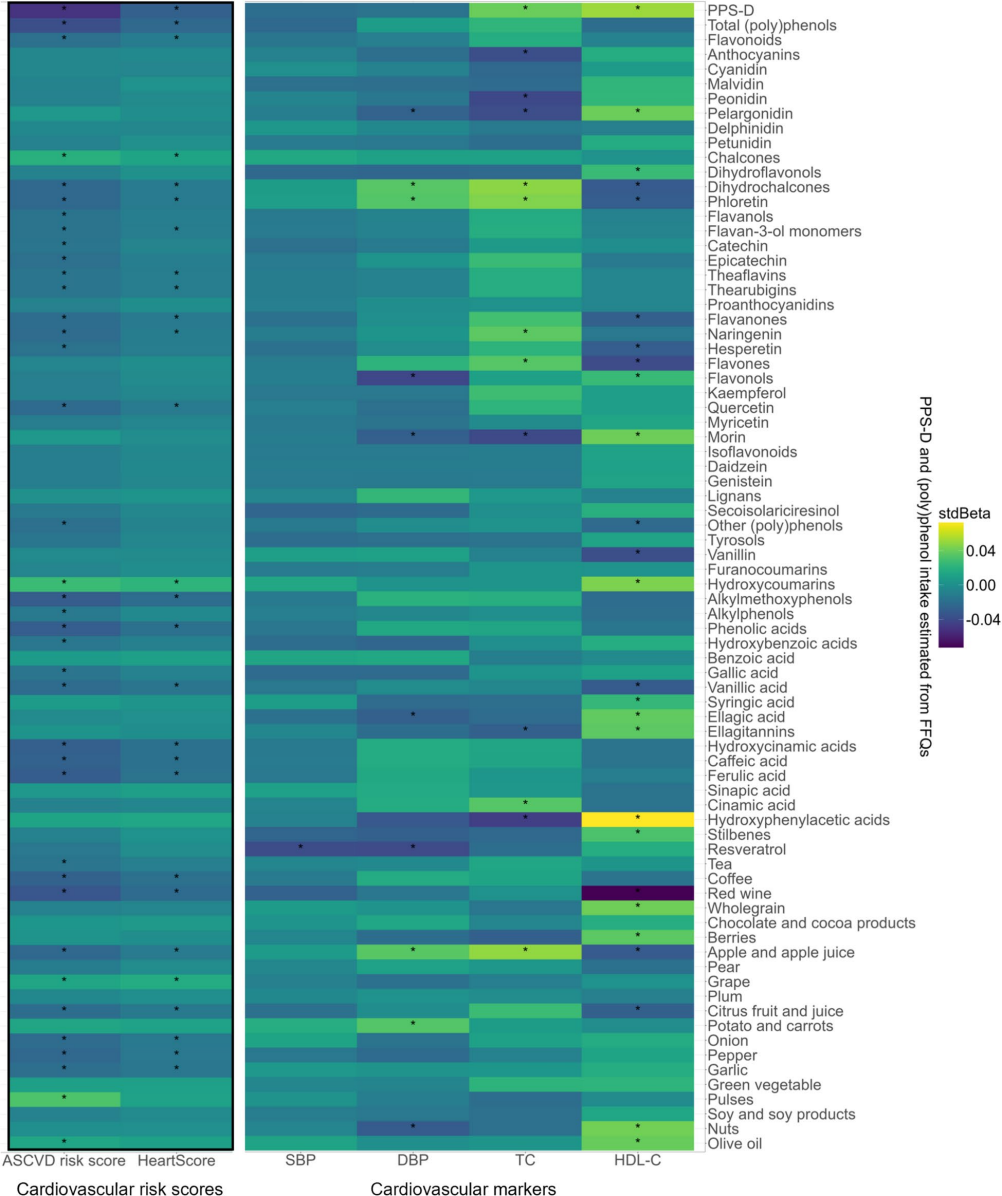
Association between cardiovascular health and (poly)phenol intake in the TwinsUK cohort adjusting for relatedness, baseline PPS-D or (poly)phenol intake level, age, sex, ethnicity, BMI, fibre, energy, alcohol, and sodium intake (*n* = 3,110) using linear mixed models. The colour scale indicates the effect (stdBeta) of each (poly)phenol intake level or PPS-D on cardiovascular health outcomes and markers. Red and blue illustrate positive and negative effects, and colour intensity represents the degree of the effects. The asterisks showed significance (*: all FDR adjusted *p* < 0.05). PPS-D, (poly)phenol-rich dietary score; SBP, systolic blood pressure; DBP, diastolic blood pressure; TC, total cholesterol; HDL-C, high-density lipoproteins cholesterol; ASCVD risk score, atherosclerotic cardiovascular disease risk score

The association between cardiovascular risk, PPS-D and (poly)phenol intake levels is shown in Fig. 3. As for the ASCVD risk score, 24 of the 57 (poly)phenol groups and 8 of the 20 (poly)phenol-rich food items showed negative associations, whereas 16 (poly)phenol groups and 7 (poly)phenol-rich food items showed negative associations with HeartScore (all FDR-adjusted *p* < 0.05). PPS-D presented the strongest effect on ASCVD risk score (stdBeta: − 0.05, 95% CI: − 0.07, − 0.04), followed by the effect of total (poly)phenols (stdBeta: − 0.04, 95% CI: − 0.05, − 0.02) and red wine on ASCVD risk score (stdBeta: − 0.03, 95% CI: − 0.05, − 0.02), respectively (all FDR-adjusted *p* < 0.01).

### PPS-M, circulating (poly)phenol metabolites and CVD risk

In the subset of 200 volunteers with available urinary metabolites, we conducted the analysis of (poly)phenol exposure on cardiovascular health with objective measurements, including biomarkers and PPS-M. The PPS-M was established following the methodologies developed previously with the same selected metabolites and predefined penalised weight [24]. The dose–response relationship of scaled PPS-M and cardiovascular health measurements is shown in Additional file 1: Fig. S4. The *Y*-axis represents the residual of each cardiovascular measurement, regressing out family relatedness, age, BMI, fibre, energy, alcohol and sodium intake. A positive association was observed between the PPS-M and HDL-C levels, while inverse associations were identified for SBP, DBP, ASCVD risk score, and HeartScore, suggesting that a higher PPS-M was correlated with elevated HDL-C and lower blood pressure, ASCVD risk, and HeartScore. However, none of these associations reached statistical significance (FDR-adjusted *p* > 0.05, Additional file 2: Table S4).

**Fig. 4:**
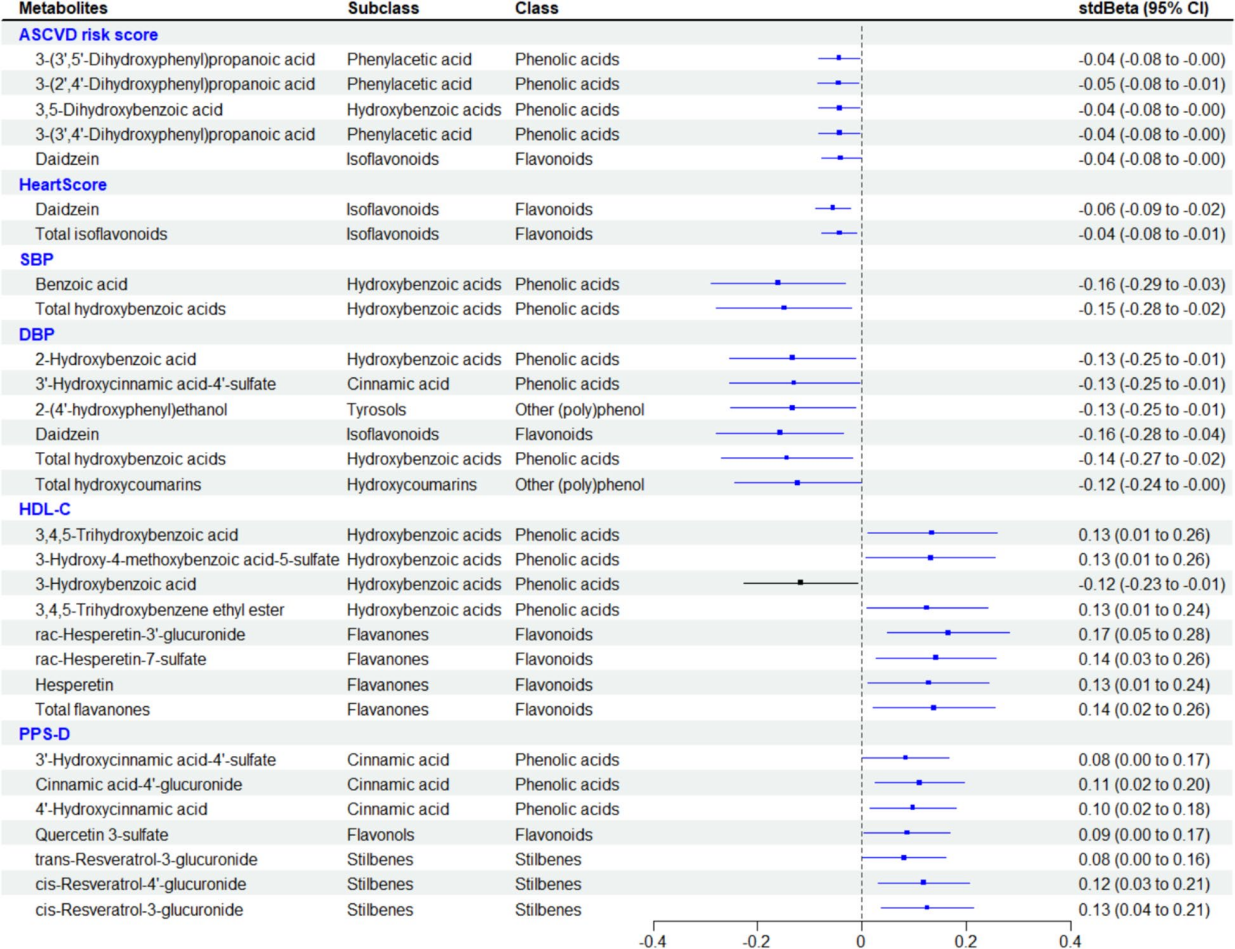
Forest plot of significant associations (stdBeta) between urinary (poly)phenol metabolites, cardiovascular health, and PPS-D in the TwinsUK cohort (*n* = 200) adjusting for family relatedness, age, BMI, fibre, energy, alcohol, and sodium intake in linear mixed models. Only metabolites with significant associations are presented in this figure (FDR-adjusted *p* < 0.05). PPS-D, (poly)phenol-rich dietary score; SBP, systolic blood pressure; DBP, diastolic blood pressure; HDL-C, high-density lipoproteins cholesterol; ASCVD risk score, atherosclerotic cardiovascular disease risk score

The cross-sectional analysis of the associations between urinary metabolites and PPS-D/PPS-M and CVD risk measurements is shown in Additional file 1: Fig. S5. Significant associations between urinary metabolites, PPS-D and CVD risk markers were selected and presented in Fig. 4. Positive associations with the PPS-M metabolic signature were observed for 127 of the 141 urinary metabolites, with stdBeta ranging from 0.14 (95% CI: 0.01, 0.03) for enterodiol to 0.99 (95% CI: 0.98, 1.01) for total hippuric acids (Additional file 1: Fig. S5). Of these, 7 metabolites passed correction for multiple testing, significantly associating with PPS-D, with stdBeta ranging from 0.08 (95% CI: 0.001, 0.16) for trans-resveratrol-3-glucuronide to 0.13 (95% CI: 0.04, 0.21) for cis-resveratrol-3-glucuronide (all FDR adjusted *p* < 0.05) (Fig. 4). As for CVD risk scores, significant negative associations were found between HeartScore and the urinary metabolites daidzein (stdBeta (95% CI): − 0.06 (− 0.09, − 0.02)), as well as total isoflavonoids (stdBeta (95% CI): − 0.04 (− 0.08, − 0.01)). The ASCVD risk score was significantly associated with 5 metabolites, including 3-(3′,5′-dihydroxyphenyl)propanoic acid, 3-(2′,4′-dihydroxyphenyl)propanoic acid, 3,5-dihydroxybenzoic acid, 3-(3′,4′-dihydroxyphenyl)propanoic acid, and daidzein (stdBeta (95% CI): − 0.04 (− 0.08, − 0.04), all FDR adjusted *p* values < 0.05). SBP was negatively associated with benzoic acids (− 0.16 (− 0.29, − 0.03)) and total hydroxybenzoic acids (− 0.15 (− 0.28, − 0.02)), whereas DBP was associated with 6 metabolites, including 2-hydroxybenzoic acid, 3′-hydroxycinnamic acid-4′-sulphate, 2-(4′-hydroxyphenyl)ethanol, daidzein, total hydroxybenzoic acids, and total hydroxycoumarins with stdBeta ranging from − 0.16 (− 0.28, − 0.04) to − 0.12 (− 0.24, − 0.003) (all FDR adjusted *p* values < 0.05). HDL-C was positively associated with 7 metabolites, including 3-hydroxy-4-methoxybenzoic acid-5-sulphate, 3,4,5-trihydroxybenzoic acid, 3,4,5-trihydroxybenzene ethyl ester from the hydroxybenzoic acids subclass, and rac-hesperetin-3′-glucuronide, rac-hesperetin-7-sulphate, hesperetin, and total flavanones from the flavanone subclass (all FDR adjusted *p* values < 0.05).

## Discussion

In this large study, we report that adherence to (poly)phenol-rich diets is negatively correlated to longitudinal cardiovascular risk scores, ASCVD and HeartScore, with PPS-D reflecting a stronger association than (poly)phenol intake or individual (poly)phenol-rich foods. These results were replicated through urinary flavonoid and phenolic acid metabolite analysis, which showed significant associations with ASCVD risk score, HeartScore, DBP, and HDL-C. These findings highlight the potential long-term cardiovascular benefits of (poly)phenol intake, particularly phenolic acids, flavonoids, and tyrosols.

Total (poly)phenol intake, derived from FFQ data, showed the strongest association with ASCVD and HeartScore compared to individual or subgroup intakes, highlighting its robustness in association with cardiovascular risk scores. However, capturing dietary (poly)phenol intake remains challenging due to the vast diversity of over 8000 identified plant (poly)phenols so far [73], with only 715 listed in databases like Phenol-Explorer [30]. The apriori PPS dietary score provides a comprehensive assessment of adherence to (poly)phenol-rich diets without relying on (poly)phenol composition databases. It is derived from 20 predefined food groups known to be rich in (poly)phenols, including tea, coffee, red wine, whole grains, chocolate, berries and olive oil [18]. PPS-D demonstrated a stronger association with cardiovascular risk scores than total (poly)phenol intake, highlighting its effectiveness in ranking participants by their habitual (poly)phenol intake. Among PPS-D components, tea, coffee, and red wine—the primary sources of phenolic acids, flavonoids, other (poly)phenols, and stilbenes—were particularly associated with more favourable cardiovascular profiles, as were onions, peppers, garlic, nuts, whole grains, and berries. Potatoes, despite being less (poly)phenol-dense, are included in the PPS-D [18] due to their high consumption in the UK and their contribution to total (poly)phenol intake [74]. It is classified as a less healthy food in the graded dietary pattern plant-based dietary index (PDI), where higher intake of less healthy plant foods has been associated with a 16.0% increased risk of type 2 diabetes [75, 76]. Consistently, the food groups of potatoes and carrots did not show any significant associations with DBP, possibly due to the inclusion of low-quality food such as fried potato chips and salad potatoes (with mayonnaise). Similarly, baked beans, included in the pulses group and often high in sugar and salt, may explain the positive association with ASCVD risk score. Fruit juice, another less healthy plant beverage in the PDI grading system due to loss of fibre during processing, higher sugar bioavailability, and reduced phytochemical levels, may also contribute to inconsistent findings. Although fruit and vegetable intake, especially apple and citrus fruits, are cardioprotective [77], combining whole fruits and juices in one category may explain the contradictory associations observed in apple/citrus and juice with cardiovascular markers. As a result, the protective effects on ASCVD risk score and HeartScore may primarily arise from whole fruits, and the adverse associations in our analysis of apple/citrus and juice with cardiovascular markers (DBP, TC, HDL-C) may largely be attributable to fruit juices.

Metabolomics profiling provides a comprehensive analytical approach for identifying biomarkers linked to specific dietary patterns [21, 24]. This methodology enables metabolic signature as a more objective and precise measure of adherence to (poly)phenol-rich diets, providing an advantage over PPS-D, which relies on traditional dietary assessment tools and may be subject to reporting biases. However, the PPS-M, derived from a representative panel of 51 metabolites across five (poly)phenol classes, was not significantly correlated with elevated HDL-C and declined blood pressure, ASCVD risk score, and HeartScore. Several factors may explain this finding. Despite the 200 participants for metabolic signature being enough for the sample size, the PPS sample size of 3110 participants might provide a higher statistical power to detect the effects of (poly)phenol-rich diets on cardiovascular risk. In addition, multiple biofluids, such as spot urine, 24-h urine, and plasma, are commonly used for biomarker analysis [21]. The PPS-M was developed using 24-h urine and validated with spot urine and plasma samples [24]. While 24-h urine provides a more comprehensive metabolite profile and captures compounds with varying half-lives, it is more burdensome for participants, which is why spot urine was used in this study [24, 78]. This choice may have attenuated the associations with cardiovascular risk. Plasma samples are also valuable in studies focused on cardiovascular health due to their rich content of lipid-soluble, metabolically active compounds [78]. However, urine samples are better suited for assessing dietary (poly)phenol intake, as they capture a larger range of food-derived phytochemicals and secondary metabolites [79]. Most metabolites would be excreted faster in urine samples, making it an effective biomarker for food intake [21]. Therefore, using multiple biofluids to replicate the PPS-M’s impact on cardiovascular health would provide a more robust analysis.

Total flavonoids, phenolic acids, and other (poly)phenols were inversely associated with cardiovascular risk scores, with urinary metabolites identifying such protective effects. Here, phenolic acids and flavonoids represented two main (poly)phenol classes with the most significant associations with favourable outcomes, i.e. DBP, TC, HDL-C, ASCVD risk score, and HeartScore, consistent with their cardioprotective and anti-atherosclerotic effects [8, 9, 80, 81]. This FFQ-derived result was also replicated through urinary metabolites, such as the negative associations between ASCVD score and daidzein (flavonoids), 3,5-dihydroxybenzoic acid and 3-(3′,5′/2′,4′/3′,4′-dihydroxyphenyl)propanoic acid (phenolic acids). As the major food sources of phenolic acids and flavonoids, higher tea and coffee intake were associated with lower CVD risk, indicating that a 100 g d^−1^ (0.53 cup per d) increase in tea and coffee intake was associated with a 0.75% and 1.3% reduction in ASCVD risk score, respectively, supporting prior findings on their cardioprotective effect [82, 83]. Moreover, other (poly)phenols, including compounds with less typical chemical structures, such as tyrosols and hydroxycoumarins [84], also presented protective effects on ASCVD risk score, HeartScore, and HDL-C. This was further replicated through the association between DBP and urinary total hydroxycoumarins and 2-(4′-hydroxyphenyl)ethanol (tyrosols), consistent with the findings that dietary tyrosols are associated with a lower prevalence of atherosclerosis [85]. In contrast, stilbenes and lignans, having the lowest intake among all classes, did not show significant associations with CVD risk scores, except for a protective effect of resveratrol on SBP and DBP and stilbenes on HDL-C. In agreement with the data derived from questionnaires, no significant associations were observed between urinary stilbene and lignan and CVD outcomes, which may be due to the low consumption in our cohort.

Here, the longitudinal data of both PPS-D/(poly)phenol intake and cardiovascular markers enabled the exploration of the relationship between (poly)phenol consumption and cardiovascular health outcomes, enhancing a more reliable and generalisable finding for a wider population. Health awareness increases with age, leading to a more (poly)phenol-rich diet, reflected by higher PPS dietary scores. ASCVD risk scores in our study increased from baseline (mean 0.03 ± 0.04) to follow-up (mean 0.10 ± 0.12) within the categories of low risk (< 5.0%) to intermediate risk categories (7.5–19.9%) [58]. The present study found that a 10 unit increase in PPS-D was associated with an 8.5% reduction of ASCVD risk score, and a 100 mg day^−1^ increase in total (poly)phenol intake was associated with a 0.6% reduction of ASCVD risk score, which aligned with the evidence on their cardioprotective effects [86]. Although cardiovascular risk increases with age, our study indicates that maintaining a high (poly)phenol-rich diet may substantially slow its progression, highlighting the long-term protective effects of (poly)phenol-rich diets in older adults.

This study is the first to comprehensively assess (poly)phenol intake and cardiovascular health in the UK population using the novel PPS-D dietary pattern, supported by complementary approaches including PPS-M metabolic signatures, urinary (poly)phenol metabolites, and FFQ-derived (poly)phenol intake estimates. While FFQs remain the most widely used dietary assessment tool in large observational studies due to their low participant burden and ability to capture long-term intake [13], the EPIC-Norfolk FFQ was not designed for estimating (poly)phenol intake and does not include key (poly)phenol-rich foods such as blueberries [18, 19]. Future studies require the use of FFQs designed to estimate (poly)phenol intake based on local UK dietary habits [87]. The predominant white, middle-aged female participants limited the generalizability of this study, and the unaccounted confounders, such as physical activity, menopausal status, and in particular medication use, may also affect CVD outcomes and interfere with (poly)phenol metabolism. Furthermore, the biospecimen collection was restricted to spot urine from 200 participants, and the adjustment by creatinine may also introduce bias since creatinine has been found to vary by factors such as age, sex, and race/ethnicity [88]. The collection of 24 h urine might be a promising choice as such an adjustment is not required, and absolute quantification is possible. Larger-scale intervention studies with biospecimens across various sample types are warranted to confirm these findings.

## Conclusions

In conclusion, longitudinal data indicate that higher adherence to (poly)phenol-rich diets is associated with lower long-term cardiovascular risk in predominantly middle-aged women. The PPS dietary score, reflecting adherence to (poly)phenol-rich diets, showed the strongest association with CVD risk compared to individual (poly)phenol intakes or specific (poly)phenol-rich food sources. The cardioprotective effects of flavonoids, phenolic acids, and tyrosols, estimated from the EPIC-Norfolk FFQ, were further confirmed through urinary metabolite analysis within the same (poly)phenol classes. Nevertheless, additional dietary intervention studies are needed to validate these associations using biomarkers, providing stronger, evidence-based guidance on the benefits of (poly)phenol-rich diets for cardiovascular health.

## Supplementary Information

Additional file 1.

Additional file 2.

## Data Availability

The data used in this study are held by the Department of Twin Research at King’s College London. The data can be released to bona fide researchers using our normal procedures overseen by the Wellcome Trust and its guidelines as part of our core funding (https://twinsuk.ac.uk/resources-for-researchers/access-our-data/).
