# Supplementary material for: Higher adherence to (poly)phenol-rich diet is associated with lower CVD risk in the TwinsUK cohort
Source: BMC Med. 2025 Nov 27;23:645. doi: 10.1186/s12916-025-04481-5 (PMC12659045; doi:10.1186/s12916-025-04481-5)
Supplement: Supplementary file 1 — Additional file 1. [file 12916_2025_4481_MOESM1_ESM.docx]

**Higher adherence to (poly)phenol-rich diet is associated with lower CVD risk in the TwinsUK cohort**

Yong Li ^1^, Xinyu Yan ^2^, Yifan Xu ^1^, Robert Pope ^2^, Tim D Spector ^2^, Mario Falchi ^2^, Claire J Steves ^2^, Jordana T Bell ^2^, Kerrin S Small ^2^, Cristina Menni ^2,3^, Rachel Gibson ^1^, Ana Rodriguez-Mateos ^1, *^

^1^ Department of Nutritional Sciences, School of Life Course and Population Sciences, Faculty of Life Sciences and Medicine, King's College London, London, UK

^2^ Department of Twin Research and Genetic Epidemiology, School of Life Course and Population Sciences, Faculty of Life Sciences and Medicine, King's College London, London, UK

^3^ Department of Pathophysiology and Transplantation, Università Degli Studi di Milano, Milan, Italy

^*^ Author to whom correspondence should be addressed.

**Table of Content**

**Figure S1** The change of (poly)phenol intake level from baseline to follow-up time adjusted for relatedness, baseline age of FFQ, and energy intake in this TwinsUK population (n = 3110)

**Figure S2** Correlation between FFQs estimated (poly)phenol intake and PPS-D and (poly)phenol-rich food items involved in the TwinsUK cohort adjusting for family relatedness and energy intake (n = 3110).

**Figure S3** The change of cardiovascular health markers and risk scores from baseline to follow-up time adjusted for relatedness, sex, ethnicity, baseline age of cardiovascular measurement, BMI, alcohol, fibre, sodium and energy intake in this TwinsUK population (a) (n = 3110); Visualisation of the dose-response relationship of (b) the total (poly)phenol intake and (c) PPS-D with cardiovascular health outcomes (n = 3110).

**Figure S4** Visualisation of the dose-response relationship of scaled PPS-M (PPS metabolic signature) and cardiovascular health measurements (n = 200).

**Figure S5** Association between urinary (poly)phenol metabolites, PPS-D/-M, and cardiovascular health in the TwinsUK cohort adjusting for family relatedness, age, BMI, fibre, energy, alcohol, and sodium intake (n = 200).


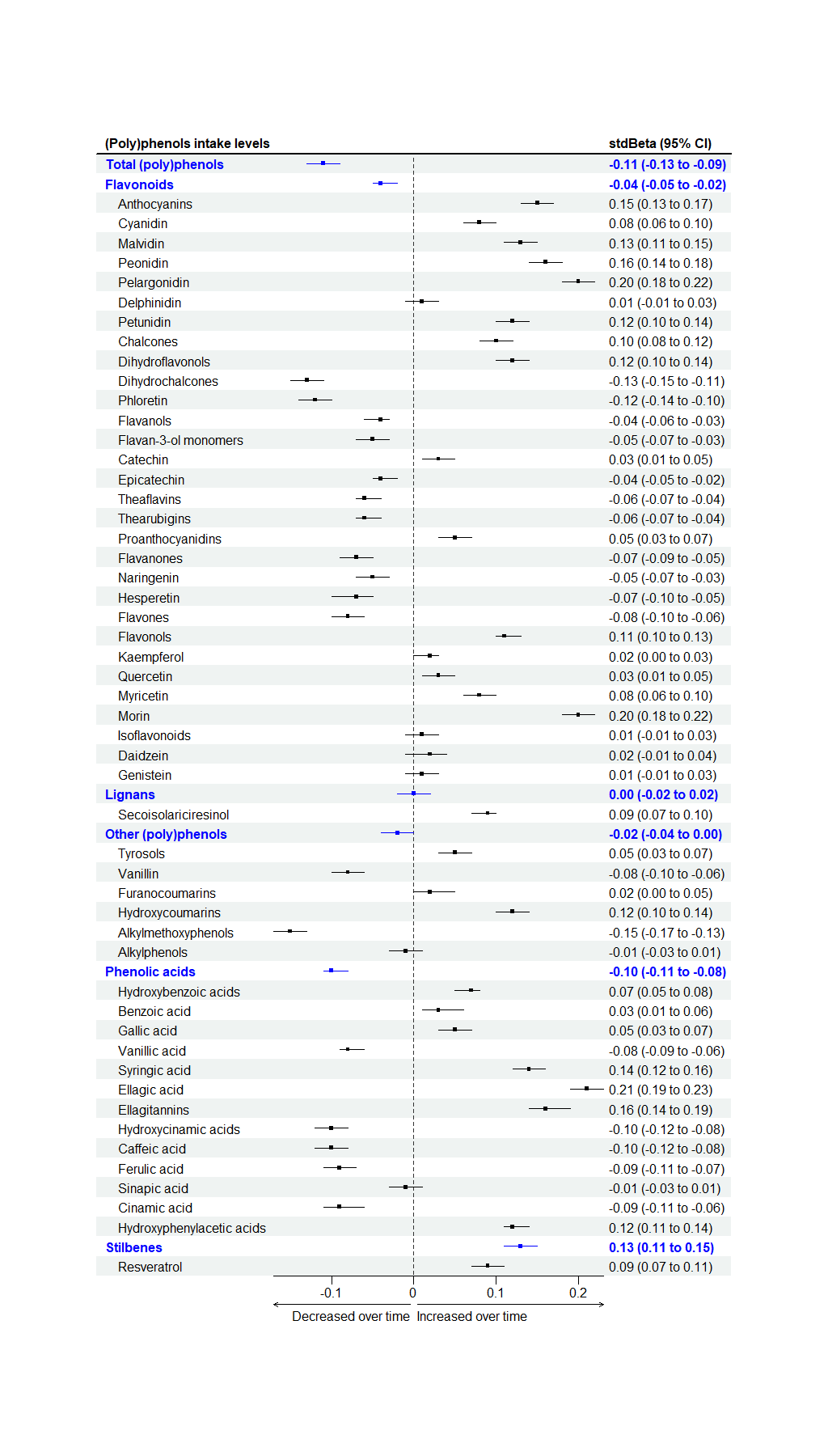


**Figure S1. The change of (poly)phenol intake level from baseline to follow-up time adjusted for relatedness, baseline age of FFQ, and energy intake in this TwinsUK population (n = 3110)**

**

**

**Figure S2. Correlation between FFQs estimated (poly)phenol intake and PPS-D and (poly)phenol-rich food items involved in the TwinsUK cohort adjusting for family relatedness and energy intake (n = 3110).** The colour scale indicates the effect (stdBeta) of each (poly)phenol intake level on PPS-D. Red and blue illustrate positive and negative effects, and colour intensity represents the degree of the effects. The asterisks showed significance (*: all FDR adjusted *p* < 0.05). PPS-D, (poly)phenol-rich dietary score.


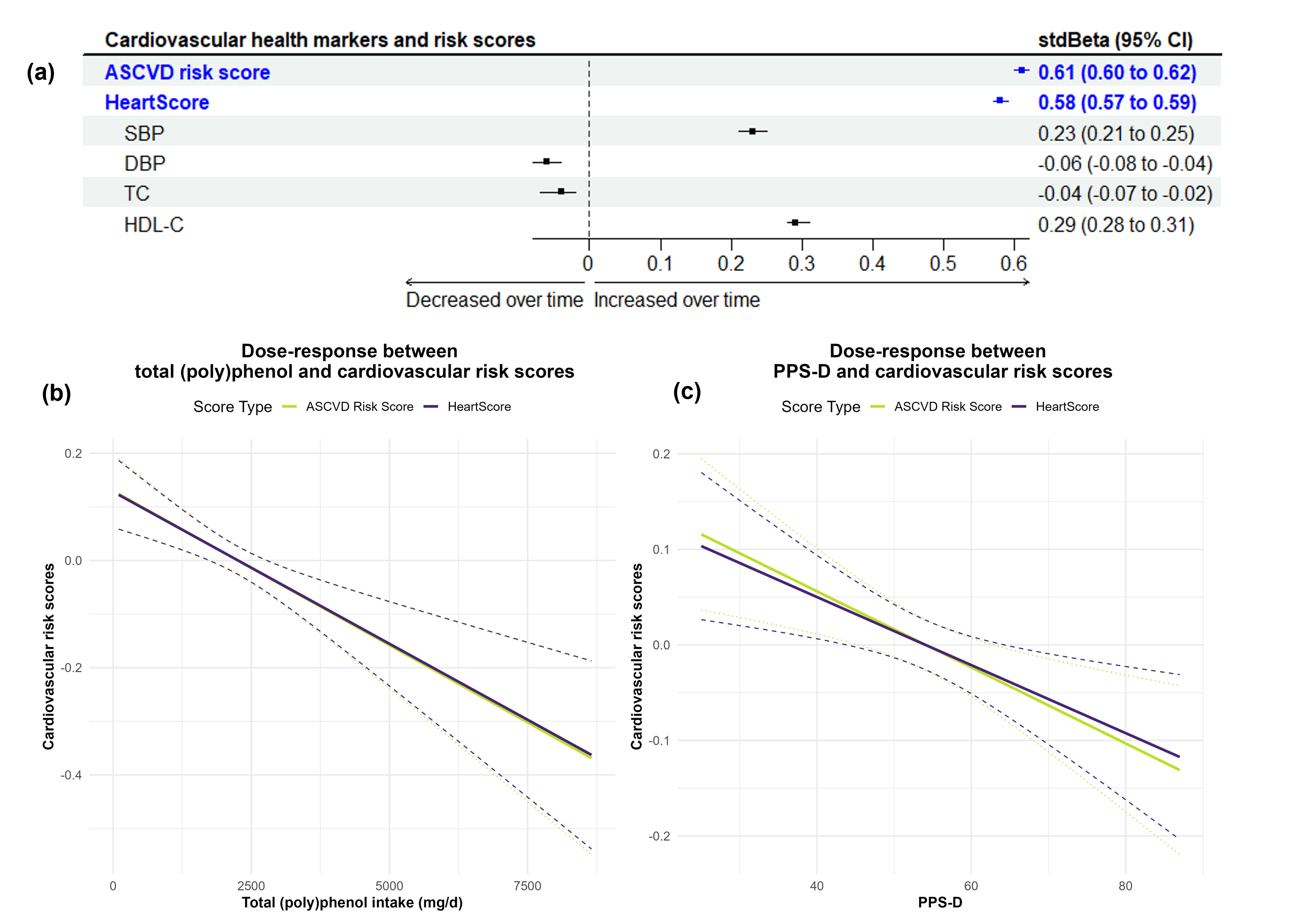


**Figure S3**. The change of cardiovascular health markers and risk scores from baseline to follow-up time adjusted for relatedness, sex, ethnicity, baseline age of cardiovascular measurement, BMI, alcohol, fibre, sodium and energy intake in this TwinsUK population (a) (n = 3110), Visualisation of the dose-response relationship of (b) the total (poly)phenol intake and (c) PPS-D with cardiovascular health outcomes (n = 3110); ASCVD risk score and HeartScore were both scaled and centred in Figure (b) and (c). BMI, body mass index; SBP, systolic blood pressure; DBP, diastolic blood pressure; TC, total cholesterol; HDL-C, high-density lipoproteins cholesterol; ASCVD risk score, atherosclerotic cardiovascular disease risk score.


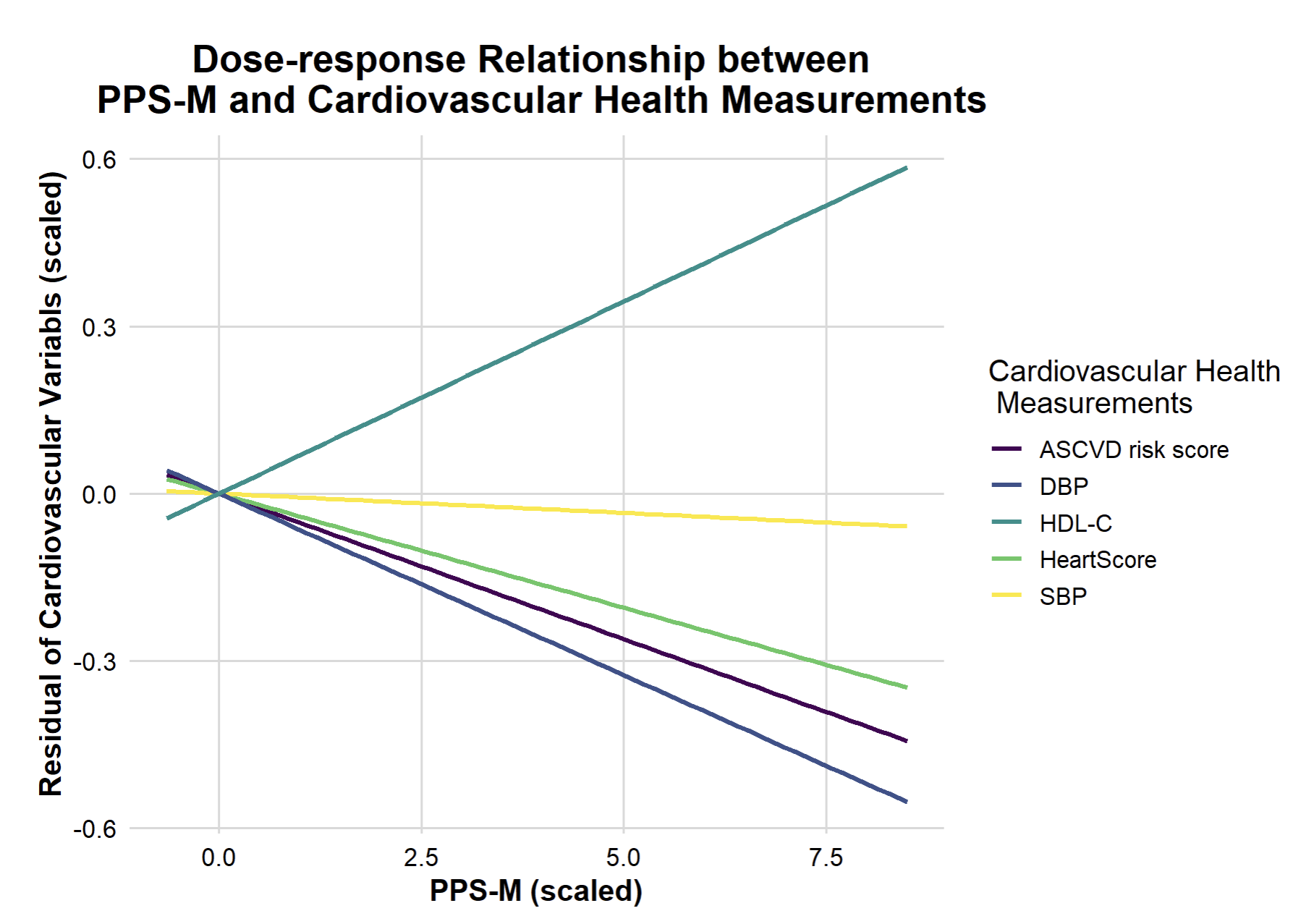


**Figure S4**. Visualisation of the dose-response relationship of scaled PPS-M (PPS metabolic signature) and cardiovascular health measurements (n = 200). Age, BMI, fibre, energy, alcohol, sodium intake and family-relatedness were regressed out for each cardiovascular measurement. PPS-M, metabolic signature of (poly)phenol-rich dietary score; SBP, systolic blood pressure; DBP, diastolic blood pressure; HDL-C, high-density lipoproteins cholesterol; ASCVD risk score, atherosclerotic cardiovascular disease risk score.


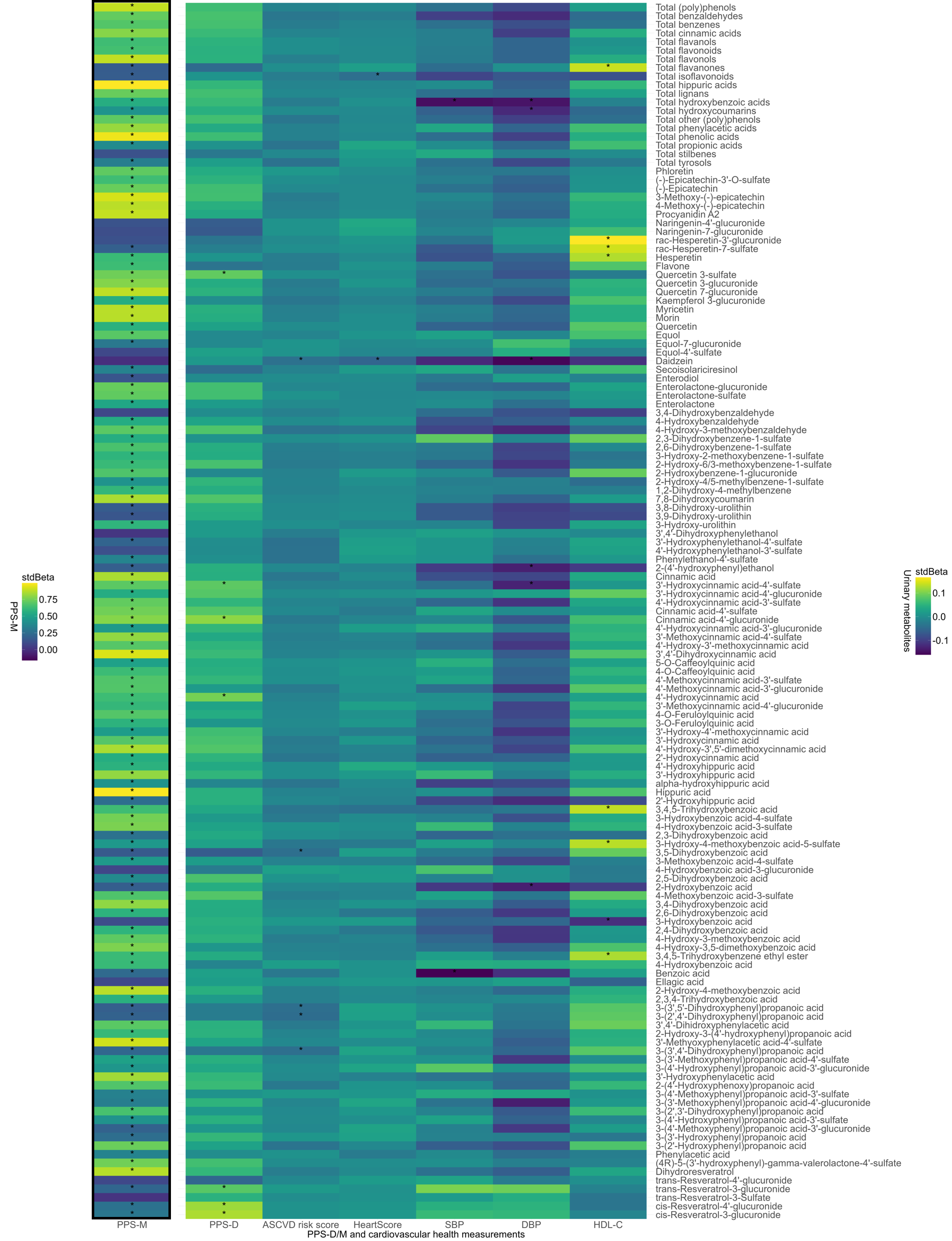


**Figure S5**. Association between urinary (poly)phenol metabolites, PPS-D/-M, and cardiovascular health in the TwinsUK cohort adjusting for family relatedness, age, BMI, fibre, energy, alcohol, and sodium intake (n = 200, cross-sectional data). The colour scale indicates the effect (stdBeta) of each urinary (poly)phenol metabolite on PPS-D/-M and cardiovascular health outcomes and markers. Red and blue illustrate positive and negative effects, and colour intensity represents the degree of the effects. The asterisks showed significance (*: all FDR adjusted *p* < 0.05). PPS-D/-M, (poly)phenol-rich dietary score and metabolic signature of (poly)phenol-rich dietary score; SBP, systolic blood pressure; DBP, diastolic blood pressure; HDL-C, high-density lipoproteins cholesterol; ASCVD risk score, atherosclerotic cardiovascular disease risk score.
