# Supplementary material for: Higher adherence to (poly)phenol-rich diet is associated with lower CVD risk in the TwinsUK cohort
Source: BMC Med. 2025 Nov 27;23:645. doi: 10.1186/s12916-025-04481-5 (PMC12659045; doi:10.1186/s12916-025-04481-5)
Supplement: Supplementary file 2 — Additional file 2. [file 12916_2025_4481_MOESM2_ESM.docx]

**Higher adherence to (poly)phenol-rich diet is associated with lower CVD risk in the TwinsUK cohort**

Yong Li ^1^, Xinyu Yan ^2^, Yifan Xu ^1^, Robert Pope ^2^, Tim D Spector ^2^, Mario Falchi ^2^, Claire J Steves ^2^, Jordana T Bell ^2^, Kerrin S Small ^2^, Cristina Menni ^2,3^, Rachel Gibson ^1^, Ana Rodriguez-Mateos ^1, *^

^1^ Department of Nutritional Sciences, School of Life Course and Population Sciences, Faculty of Life Sciences and Medicine, King's College London, London, UK

^2^ Department of Twin Research and Genetic Epidemiology, School of Life Course and Population Sciences, Faculty of Life Sciences and Medicine, King's College London, London, UK

^3^ Department of Pathophysiology and Transplantation, Università Degli Studi di Milano, Milan, Italy

^*^ Author to whom correspondence should be addressed.

**Table of Content**

**Table S1** (Poly)phenol-rich food intake of the study population measured by food frequency questionnaires in baseline and follow-up

**Table S2** (Poly)phenol intake classes and subclasses estimated from EPIC-Norfolk FFQs and their food source in baseline (mg/d)

**Table S3** (Poly)phenol intake classes and subclasses estimated from EPIC-Norfolk FFQs and their food source in follow-up (mg/d)

**Table S4** Association between PPS-M and cardiometabolic health measurements

**Table S1 (Poly)phenol-rich food intake of the study population measured by food frequency questionnaires in baseline and follow-up**

| (Poly)phenols and food items | Baseline | | | Follow-up | | |
| --- | --- | --- | --- | --- | --- | --- |
|  | Mean (SD) (g/d) | Contribution to total (poly)phenol-rich food items (%) | Contribution to total (poly)phenol intake (%) | Mean (SD) (g/d) | Contribution to total (poly)phenol-rich food items (%) | Contribution to total (poly)phenol intake (%) |
| Total (poly)phenol intake | 2364.3 (996.3) | -- | 100 | 2136.2 (911.3) | -- | 100 |
| PPS | 53.7 (10.4) | -- | 96 | 54.3 (10.4) | -- | 95.5 |
| (Poly)phenol-rich food items involved in PPS | | | | | | |
| Tea | 546.3 (375.0) | 36.8 | 39.3 | 518.7 (363.5) | 37.7 | 40.1 |
| Coffee | 380.3 (361.5) | 25.6 | 44.0 | 319.6 (316.1) | 23.3 | 40.9 |
| Red wine | 48.7 (82.9) | 3.3 | 0.8 | 29.3 (66.2) | 2.1 | 1.2 |
| Whole grains | 81.6 (67.5) | 5.5 | 1.0 | 96.8 (73.7) | 7.0 | 1.0 |
| Chocolate and cocoa products | 3.3 (5.8) | 0.2 | 0.8 | 3.9 (7.0) | 0.3 | 1.5 |
| Berries | 7.0 (9.6) | 0.5 | 0.7 | 10.2 (13.4) | 0.7 | 1.0 |
| Apple and apple juice | 86.5 (74.6) | 5.8 | 4.3 | 65.6 (62.4) | 4.8 | 3.7 |
| Pear | 23.0 (37.4) | 1.5 | 0.1 | 22.2 (34.9) | 1.6 | 0.1 |
| Grape | 11.6 (19.4) | 0.8 | 0.4 | 16.3 (24.8) | 1.2 | 0.7 |
| Plum | 2.9 (4.9) | 0.2 | 0.4 | 2.8 (4.9) | 0.2 | 0.5 |
| Citrus fruit and juice | 76.4 (76.2) | 5.1 | 1.7 | 63.1 (70.0) | 4.6 | 1.5 |
| Potato and carrots | 108.8 (55.9) | 7.3 | 1.2 | 99.5 (56.3) | 7.2 | 1.2 |
| Onion | 11.7 (10.5) | 0.8 | 0.1 | 12.2 (11.3) | 0.9 | 0.1 |
| Pepper | 4.9 (6.3) | 0.3 | 0.0 | 5.5 (6.9) | 0.4 | 0.0 |
| Garlic | 1.1 (1.6) | 0.1 | 0.1 | 1.1 (1.7) | 0.1 | 0.1 |
| Green vegetables | 29.0 (28.8) | 1.9 | 0.4 | 32.8 (32.7) | 2.4 | 0.6 |
| Pulses | 52.4 (37.3) | 3.5 | 0.4 | 57.7 (39.8) | 4.2 | 0.6 |
| Soy and soy products | 5.8 (33.7) | 0.4 | 0.1 | 8.5 (45.9) | 0.6 | 0.1 |
| Nuts | 4.3 (8.0) | 0.3 | 0.3 | 7.6 (12.2) | 0.6 | 0.7 |
| Olive oil | 0.8 (2.4) | 0.1 | 0.0 | 1.2 (3.2) | 0.1 | 0.0 |

**Table S2 (Poly)phenol intake classes and subclasses estimated from EPIC-Norfolk FFQs and their food source in baseline (mg/d)**

| (Poly)phenols (mg/d) | Mean (SD) | Median (IQR) | % | EPIC-Norfolk FFQs estimated (poly)phenol food sources (% to total) |
| --- | --- | --- | --- | --- |
| **Total (poly)phenols** | 2364.3 (996.3) | 2350.9 (1204.2) | 100 | Coffee (44.0%), Black tea (39.3%), Apples (3.4%), Oranges (0.9%), Tomatoes (0.8%), Apple juice (0.7%), Orange juice (0.7%), Old potatoes (0.6%), Red wine (0.6%), Grapes (0.4%) |
| **Flavonoids** | 1082.4 (603.1) | 1006.4 (1025) | 45.8 | Black tea (76.7%), Apples (6.4%), Oranges (1.9%), Tomatoes (1.6%), Apple juice (1.3%), Orange juice (1.2%), Red wine (0.9%), Grapes (0.9%), Drinking chocolate powder (0.8%), Dark chocolates (0.8%) |
| Anthocyanins | 12.4 (12.8) | 8.8 (11.3) | 0.5 | Fruit drink/squash (26.7%), Red wine (18.9%), Strawberries (12.9%), Raspberries (10.3%), Fruit jam (9.7%), Plums (5.7%), Orange juice (3.8%), Apples (2.8%), Mousse (2.1%) |
| Cyanidin | 4.4 (3.9) | 3.5 (3.7) | 0.2 | Raspberries (26.2%), Fruit drink/squash (22.9%), Plums (14.2%), Orange juice (10.7%), Apples (8%), Fruit jam (5.6%), Cereal bars, with fruit or nuts (2.8%), Strawberries (2.4%), Peaches (1.2%) |
| Malvidin | 1.9 (5.2) | 0.3 (0.9) | 0.1 | Red wine (85.0%), Fruit jam (10.6%), Fruit cocktail (1.8%), Raspberries (0.8%) |
| Peonidin | 0.5 (0.8) | 0.2 (0.4) | 0.0 | Red wine (37.1%), Cereal bars, with fruit or nuts (26.6%), Plums (16.4%), Fruit jam (15.5%), Fruit drink/squash (1.4%), Fruit cocktail (0.8%) |
| Pelargonidin | 2.2 (2.4) | 1.7 (2.3) | 0.1 | Strawberries (66.7%), Mousse (11.1%), Fruit jam (7.9%), Whole milk yogurt, fruit (7.6%), Raspberries (4.9%) |
| Delphinidin | 2.8 (5.9) | 0.9 (2.3) | 0.1 | Fruit drink/squash (79.1%), Fruit jam (13%), Red wine (6%), Cereal bars, with fruit or nuts (1.0%) |
| Petunidin | 0.5 (0.8) | 0.2 (0.4) | 0.0 | Red wine (53.2%), Fruit jam (31.4%), Fruit drink/squash (11.7%), Ice cream, non-dairy, vanilla (1.5%), Cereal bars, with fruit or nuts (1.2%) |
| Chalcones | 0.0 (0.0) | 0.0 (0.0) | 0.0 | Broad beans (92.2%), Lager (7.8%) |
| Dihydroflavonols | 0.8 (2) | 0.1 (0.4) | 0.0 | Red wine (80.3%), Rose wine (10.9%), White wine (8.9%) |
| Dihydrochalcones | 3.3 (2.9) | 2.5 (3.8) | 0.1 | Apples (71.2%), Apple juice (27.7%), Apple chutney (1.0%) |
| Phloretin | 1.0 (0.9) | 0.7 (1.2) | 0.0 | Apples (75.8%), Apple juice (23.7%), Apple chutney (0.4%) |
| Flavanols | 973 (588.2) | 897 (1026.2) | 41.2 | Black tea (83.8%), Apples (6.7%), Apple juice (1.3%), Grapes (1.0%), Drinking chocolate powder (0.9%), Dark chocolates (0.9%), Broad beans (0.8%), Plums (0.7%), Red wine (0.7%), Strawberries (0.5%) |
| Flavan-3-ol monomers | 282.1 (186.3) | 257.1 (331.0) | 11.9 | Black tea (92.5%), Apples (2.0%), Broad beans (1.5%), Apple juice (0.9%), Red wine (0.7%), Bananas (0.3%), Drinking chocolate powder (0.2%), Rose wine (0.2%), Dark chocolates (0.2%), Pears (0.2%) |
| Catechin | 19.4 (10.0) | 18.3 (14.6) | 0.8 | Black tea (66.9%), Red wine (5.8%), Broad beans (4.3%), Apples (3.7%), Bananas (3.6%), Apple juice (2.6%), Rose wine (1.5%), Drinking chocolate powder (1.4%), Strawberries (1.2%), Grapes (0.8%) |
| Epicatechin | 33.8 (16.6) | 33.2 (24.5) | 1.4 | Black tea (61.5%), Apples (14.6%), Apple juice (5.9%), Broad beans (5.7%), Red wine (1.8%), Pears (1.3%), Drinking chocolate powder (1.2%), Dark chocolates (1.1%), Mars bar (0.6%), Twix (0.6%) |
| Theaflavins | 65.6 (46.9) | 59.1 (82.8) | 2.8 | Black tea (100%) |
| Thearubigins | 428.7 (306.5) | 386.2 (540.6) | 18.1 | Black tea (100%) |
| Proanthocyanidins | 196.5 (101.1) | 183.7 (117.7) | 8.3 | Black tea (30.6%), Apples (30.4%), Apple juice (5.1%), Grapes (4.8%), Dark chocolates (4.1%), Drinking chocolate powder (4.1%), Plums (3.3%), Strawberries (2.5%), Red wine (2.5%), Broad beans (1.9%) |
| Flavanones | 36.0 (34.9) | 27.8 (41.8) | 1.5 | Oranges (56%), Orange juice (32.7%), Grapefruit (8.7%), Tomatoes (0.8%) |
| Naringenin | 9.2 (11.7) | 5.9 (9.9) | 0.4 | Oranges (54.9%), Grapefruit (33.2%), Orange juice (6.3%), Tomatoes (3.3%) |
| Hesperetin | 26.7 (26.3) | 20.9 (31.4) | 1.1 | Oranges (56.6%), Orange juice (42%), Trifle (0.5%), Grapefruit (0.3%) |
| Flavones | 5.4 (3.4) | 4.6 (3.4) | 0.2 | Brown bread (26.9%), Orange juice (15.7%), Celery (8%), Digestive biscuits, chocolate (6.4%), Semi-sweet biscuits (3.7%), Swede (3.3%), Pizza (2.9%), Pizza (2.9%), Broccoli (2.1%), Apples (1.9%) |
| Flavonols | 49.5 (23.3) | 46.3 (26.3) | 2.1 | Tomatoes (33.5%), Black tea (29.8%), Spinach (8.1%), Vegetable soup (5.6%), Onions (5%), Broccoli (3%), Apples (2.9%), Brussels sprouts (1.5%), Broad beans (1.1%), Red wine (0.7%) |
| Kaempferol | 8.7 (4.7) | 8.3 (7.3) | 0.4 | Black tea (69%), Broccoli (9.2%), Brussels sprouts (5.5%), Spinach (4.7%), Broad beans (3.5%), Leeks (2.3%), Onions (0.9%), Grapes (0.6%), Oranges (0.5%), Cauliflower (0.4%) |
| Quercetin | 16.0 (6.5) | 16.0 (9.1) | 0.7 | Black tea (46%), Onions (14.8%), Apples (9.1%), Broccoli (3.7%), Tomatoes (2.2%), Apple juice (2.2%), Spinach (1.9%), Potato crisps (1.7%), Brussels sprouts (1.4%), Chips (1.4%) |
| Myricetin | 2.3 (1.1) | 2.2 (1.6) | 0.1 | Black tea (58.8%), Mushrooms (6.8%), Broad beans (5.8%), Strawberries (5.3%), Red wine (5.3%), Fruit drink/squash (4.8%), Swede (4.3%), Mousse (0.9%), Garlic (0.8%), Leeks (0.7%) |
| Morin | 0.0 (0.0) | 0.0 (0.0) | 0.0 | Strawberries (77.5%), Mousse (12.9%), Whole milk yogurt, fruit (8.9%), Ice cream, non-dairy, vanilla (0.8%) |
| Isoflavonoids | 2.0 (5.8) | 0.1 (1.4) | 0.1 | Tofu (40.5%), Beansprouts (36.1%), Soya milk (17.1%), Vegeburger (2.3%), White bread (1.8%), Soya mince, granules (1.2%), Doughnuts, jam (0.5%) |
| Daidzein | 0.6 (2.0) | 0.0 (0.4) | 0.0 | Tofu (44.9%), Beansprouts (31.3%), Soya milk (16%), Vegeburger (3.1%), White bread (1.7%), Soya mince, granules (1.7%), Doughnuts, jam (1.0%) |
| Genistein | 1.2 (3.5) | 0.1 (0.9) | 0.1 | Beansprouts (39.4%), Tofu (36.3%), Soya milk (18.9%), White bread (2%), Vegeburger (1.8%), Soya mince, granules (0.9%) |
| **Lignans** | 2.8 (1.4) | 2.7 (1.5) | 0.1 | Old potatoes (49.2%), Potatoes, roast (7.7%), Black tea (7.6%), Broccoli (6.7%), Chips, straight cut (3.7%), Oranges (1.9%), Vegetable soup (1.7%), Cauliflower (1.2%), Muesli (1.2%), Coffee (1.2%) |
| Secoisolariciresinol | 0.4 (0.1) | 0.4 (0.2) | 0.0 | Black tea (42.3%), Old potatoes (11%), Coffee (7%), Carrots (5.4%), Red wine (3.2%), Broccoli (2.0%), Kiwi fruit (1.9%), Melon (1.8%), Potatoes (1.7%) |
| **Other (poly)phenols** | 38.1 (30.1) | 28.9 (34.6) | 1.6 | Coffee (22.4%), Bran Flakes (19.1%), Wholemeal bread (15.3%), All-Bran (11.8%), Spaghetti (6.3%), Shredded Wheat (5.6%), Brown bread (5.5%), Muesli, Swiss style (2.4%), Red wine (1.9%) |
| Tyrosols | 1.4 (2.2) | 0.7 (1.5) | 0.1 | Red wine (42.1%), Rose wine (25.1%), Olive oil (9.5%), Fat spread (8.6%), Lager (5.2%), Sherry (4.9%), White wine (3.9%) |
| Vanillin | 0.0 (0.0) | 0.0 (0.0) | 0.0 | Lager (42.9%), Sherry (31.8%), Spirits (14.1%), Vermouth (8%), Trifle (3.2%) |
| Furanocoumarins | 0.2 (0.2) | 0.2 (0.2) | 0.0 | Celery (97.9%), Lasagne (1%), Moussaka (1.0%) |
| Hydroxycoumarins | 0.1 (0.2) | 0 (0.1) | 0.0 | White wine (62.9%), Lager (35%), Sherry (1.7%) |
| Alkylmethoxyphenols | 4.3 (3.6) | 4.5 (4.9) | 0.2 | Coffee (85.2%), Fat spread (13.6%), Lager (0.8%) |
| Alkylphenols | 27.7 (28.9) | 15.8 (33.3) | 1.2 | Bran Flakes (26.2%), Wholemeal bread (21%), All-Bran (16.3%), Shredded Wheat (7.7%), Spaghetti (8.6%), Brown bread (7.6%), Muesli (3.3%), Coffee (3.1%), Shreddies (2.4%) |
| **Phenolic acids** | 1240.3 (958.2) | 1092.4 (1210.9) | 52.5 | Coffee (83.2%), Black tea (8%), Old potatoes (1.1%), Apples (0.9%), Brown rice (0.5%), Raspberries (0.4%), Cashew nuts (0.4%), Carrots, old (0.4%), White rice (0.4%), Apple juice (0.3%) |
| Hydroxybenzoic acids | 108.6 (63.0) | 99.2 (101.9) | 4.6 | Black tea (78.7%), Raspberries (4.7%), Cashew nuts (4.3%), Strawberries (1.5%), Garlic (1.3%), Cauliflower (1.2%), Red wine (1.1%), Carrots, old (0.8%), Rose wine (0.8%), Carrots, young (0.7%) |
| Benzoic acid | 0.0 (0.0) | 0.0 (0.0) | 0.0 | Drinking chocolate powder (96.7%), Fruitcake (3.1%) |
| Gallic acid | 28.4 (18.4) | 24.4 (29.7) | 1.2 | Black tea (86%), Cashew nuts (6.6%), Red wine (2.1%), Bananas (1.8%), Rose wine (1.2%) |
| Vanillic acid | 0.9 (0.5) | 0.8 (0.5) | 0.0 | Carrots (23.2%), Rose wine (14.2%), Coffee (12.7%), White rice (11.2%), Red wine (5.8%), Fruit 'n Fibre (5%), Lager (3.6%), Brown rice (3.1%), Grapefruit (2.8%) |
| Syringic acid | 0.9 (1.0) | 0.6 (0.6) | 0.0 | Walnuts (30.6%), Cauliflower (23.3%), Rose wine (21.1%), Red wine (5%), Apricots (2.9%), Fat spread (70% fat) (2.8%), Apple chutney (1.8%), Brown bread (1.3%), Drinking chocolate powder (1.2%), Semi-sweet biscuits (0.9%) |
| Ellagic acid | 5.8 (11.4) | 3.0 (6.1) | 0.2 | Cashew nuts (49.2%), Strawberries (22.4%), Raspberries (20%), Mousse (3.7%), Whole milk yogurt, fruit (2.6%), Walnuts (0.8%) |
| Ellagitannins | 3.8 (5.1) | 1.5 (3.8) | 0.2 | Raspberries (99%), Ice cream, non-dairy, vanilla (1%) |
| Hydroxycinamic acids | 1131.7 (976.8) | 980.6 (1192.4) | 47.9 | Coffee (91.1%), Black tea (1.2%), Old potatoes (1.2%), Apples (1.0%), Brown rice (0.6%), Carrots (0.6%), White rice (0.4%) |
| Caffeic acid | 297.0 (273.1) | 260.9 (333.2) | 12.6 | Coffee (96.7%), Carrots (1.1%), Broccoli (0.4%), Apples (0.3%), Old potatoes (0.3%), Tomatoes (0.3%) |
| Ferulic acid | 61.2 (41.9) | 57.6 (59.0) | 2.6 | Coffee (67.6%), Brown rice (10.9%), White rice (6.4%), Brown bread (3.6%), Fruit 'n Fibre (2.5%), Orange juice (1.8%), Rice Krispies (1.4%), Digestive biscuits, chocolate (1.1%) |
| Sinapic acid | 2.1 (1.5) | 1.7 (1.8) | 0.1 | Cauliflower (37.1%), Orange juice (21.4%), Fruit 'n Fibre (16.9%), Broccoli (12.4%), Lager (4.6%), Digestive biscuits, chocolate (2.0%), Grapefruit (1.6%), Fat spread (1.5%), Rose wine (0.8%), Red wine (0.5%) |
| Cinnamic acid | 0.2 (0.3) | 0.2 (0.3) | 0.0 | Orange juice (75.6%), Strawberries (12.7%), Coffee (6.6%), Mousse (2.1%), Whole milk yogurt, fruit (1.4%), Raspberries (1.4%) |
| Hydroxyphenylacetic acids | 0.1 (0.1) | 0.0 (0.1) | 0.0 | Red wine (38.5%), Lager (36.9%), White wine (24.2%) |
| **Stilbenes** | 0.7 (1.5) | 0.2 (0.5) | 0.0 | Red wine (69.1%), Rose wine (12.8%), White wine (12.5%), Grapes (2.8%), Strawberries (1.9%), Mousse (0.3%), Whole milk yogurt, fruit (0.2%) |
| Resveratrol | 0.2 (0.4) | 0.1 (0.2) | 0.0 | Red wine (41.8%), Rose wine (30.2%), Grapes (7.5%), Strawberries (5%), White wine (13.3%), Mousse (0.8%), Whole milk yogurt, fruit (0.6%) |

EPIC-Norfolk FFQ: European Prospective Investigation into Cancer and Nutrition food frequency questionnaire

**Table S3 (Poly)phenol intake classes and subclasses estimated from EPIC-Norfolk FFQs and their food source in follow-up (mg/d)**

| (Poly)phenols (mg/d) | Mean (SD) | Median (IQR) | % | EPIC-Norfolk FFQs estimated (poly)phenol food sources (% to total) |
| --- | --- | --- | --- | --- |
| **Total (poly)phenols** | 2136.2 (911.3) | 2094.3 (1067.0) | 100.0 | Coffee (40.9%), Black tea (40.1%), Apples (3.1%), Red wine (1.2%), Tomatoes (1.0%), Dark chocolates (0.9%), Oranges (0.9%), Grapes (0.7%), Old potatoes (0.6%), Cashew nuts (0.6%) |
| **Flavonoids** | 1021.5 (582.7) | 972.8 (1017.2) | 47.8 | Black tea (74.9%), Apples (5.5%), Dark chocolates (1.9%), Oranges (1.9%), Tomatoes (1.8%), Red wine (1.7%), Grapes (1.4%), Strawberries (1.0%), Broad beans (1.0%), Apple juice (0.8%) |
| Anthocyanins | 15.6 (14.9) | 11.5 (12.8) | 0.7 | Red wine (25.9%), Fruit drink/squash (21.5%), Strawberries (14.9%), Raspberries (12%), Fruit jam (7%), Plums (4.5%), Whole milk yogurt, fruit (2.7%), Mousse (2.0%), Orange juice (1.9%), Apples (1.8%) |
| Cyanidin | 4.8 (4.5) | 3.8 (3.8) | 0.2 | Raspberries (34.7%), Fruit drink/squash (21.1%), Plums (12.7%), Orange juice (6.1%), Apples (5.9%), Fruit jam (4.6%), Cereal bars, with fruit or nuts (4.2%), Strawberries (3.1%), Peaches (1.1%) |
| Malvidin | 3.1 (6.5) | 0.7 (2.3) | 0.1 | Red wine (90.9%), Fruit jam (5.9%), Fruit cocktail (1.1%), Raspberries (0.7%), Cereal bars, with fruit or nuts (0.6%), Ice cream, non-dairy, vanilla (0.4%) |
| Peonidin | 0.7 (0.9) | 0.3 (0.6) | 0.0 | Red wine (44%), Cereal bars, with fruit or nuts (31.8%), Plums (11.1%), Fruit jam (9.6%), Fruit drink/squash (1%), Beans, red kidney (0.9%) |
| Pelargonidin | 3.2 (3.5) | 2.3 (3.4) | 0.2 | Strawberries (67.4%), Whole milk yogurt, fruit (12%), Mousse (8.9%), Raspberries (4.9%), Fruit jam (4.9%) |
| Delphinidin | 2.9 (6.4) | 0.9 (1.7) | 0.1 | Fruit drink/squash (76.4%), Fruit jam (11.1%), Red wine (9.8%), Cereal bars, with fruit or nuts (1.6%) |
| Petunidin | 0.6 (1.0) | 0.3 (0.7) | 0.0 | Red wine (67.2%), Fruit jam (20.8%), Fruit drink/squash (8.7%), Cereal bars, with fruit or nuts (1.4%), Ice cream, non-dairy, vanilla (1.1%) |
| Chalcones | 0.0 (0.0) | 0.0 (0.0) | 0.0 | Broad beans (95.8%), Lager (4.2%) |
| Dihydroflavonols | 1.2 (2.5) | 0.3 (1.5) | 0.1 | Red wine (88.2%), White wine (11.7%), Rose wine (0.3%) |
| Dihydrochalcones | 2.5 (2.4) | 2.1 (3.3) | 0.1 | Apples (75.9%), Apple juice (22.7%), Apple chutney (1.3%), Fruit 'n Fibre (0.2%) |
| Phloretin | 0.7 (0.7) | 0.6 (1.0) | 0.0 | Apples (80%), Apple juice (19.3%), Apple chutney (0.6%), Fruit 'n Fibre (0.2%) |
| Flavanols | 913.0 (567.5) | 869.8 (1029.5) | 42.7 | Black tea (82.3%), Apples (5.8%), Dark chocolates (2.1%), Grapes (1.5%), Red wine (1.3%), Broad beans (1.0%), Apple juice (0.8%), Strawberries (0.8%), Drinking chocolate powder (0.8%), Plums (0.7%) |
| Flavan-3-ol monomers | 262.5 (178.8) | 254.5 (335.8) | 12.3 | Black tea (91.6%), Broad beans (1.9%), Apples (1.7%), Red wine (1.2%), Apple juice (0.6%), Dark chocolates (0.4%), Bananas (0.3%), Drinking chocolate powder (0.2%), Pears (0.2%), Beans, butter (0.1%) |
| Catechin | 19.2 (10.2) | 18.2 (15.2) | 0.9 | Black tea (62%), Red wine (9.9%), Broad beans (5.1%), Bananas (3.4%), Apples (3%), Strawberries (1.7%), Apple juice (1.6%), Dark chocolates (1.3%), Grapes (1.2%), Drinking chocolate powder (1.1%) |
| Epicatechin | 31.7 (16.2) | 30.6 (23.8) | 1.5 | Black tea (60.3%), Apples (12.6%), Broad beans (7.1%), Apple juice (3.9%), Red wine (3.3%), Dark chocolates (2.7%), Drinking chocolate powder (1.7%), Pears (1.3%), Raspberries (0.8%) |
| Theaflavins | 60.5 (44.9) | 59.1 (82.8) | 2.8 | Black tea (100%) |
| Thearubigins | 395.1 (293.1) | 386.2 (540.6) | 18.5 | Black tea (100%) |
| Proanthocyanidins | 195.0 (106.0) | 177.3 (119.1) | 9.1 | Black tea (28.4%), Apples (24.8%), Dark chocolates (9.5%), Grapes (6.7%), Red wine (4.2%), Strawberries (3.7%), Plums (3.3%), Drinking chocolate powder (3.2%), Apple juice (3.2%), Broad beans (2.3%) |
| Flavanones | 29.8 (32.0) | 23.8 (38.2) | 1.4 | Oranges (65.9%), Orange juice (24.7%), Grapefruit (6.2%), Tomatoes (1.1%) |
| Naringenin | 7.7 (11.1) | 4.5 (9.2) | 0.4 | Oranges (64.1%), Grapefruit (23.2%), Orange juice (4.7%), Tomatoes (4.2%), Red wine (1.5%), Vegetable soup (0.8%) |
| Hesperetin | 22 (24) | 17.4 (27.4) | 1.0 | Oranges (66.8%), Orange juice (31.7%), Trifle (0.5%) |
| Flavones | 4.5 (2.6) | 3.9 (2.9) | 0.2 | Brown bread (21.2%), Orange juice (11.7%), Celery (9.8%), Digestive biscuits, chocolate (6.5%), Pizza (6%), Swede (3.9%), Semi-sweet biscuits (3.4%), Broccoli (2.7%), Cream crackers (2.5%) |
| Flavonols | 52.8 (26.8) | 49.1 (28.6) | 2.5 | Tomatoes (33.6%), Black tea (25.7%), Spinach (11.3%), Vegetable soup (6.6%), Onions (4.8%), Broccoli (3%), Apples (2.2%), Brussels sprouts (1.4%), Broad beans (1.2%), Red wine (1.2%) |
| Kaempferol | 8.6 (4.7) | 8.1 (6.9) | 0.4 | Black tea (64.1%), Broccoli (10.1%), Spinach (7%), Brussels sprouts (5.6%), Broad beans (4.2%), Leeks (2.6%), Onions (0.9%), Grapes (0.8%), Oranges (0.5%), Cauliflower (0.4%) |
| Quercetin | 15.9 (6.6) | 15.5 (8.9) | 0.7 | Black tea (42.8%), Onions (15.5%), Apples (7.4%), Broccoli (4.1%), Spinach (2.8%), Tomatoes (2.4%), Dark chocolates (1.9%), Grapes (1.7%), Potato crisps (1.5%), Red wine (1.5%) |
| Myricetin | 2.4 (1.2) | 2.3 (1.6) | 0.1 | Black tea (52.1%), Red wine (8.7%), Strawberries (7.4%), Broad beans (6.6%), Mushrooms (6.1%), Fruit drink/squash (4.7%), Swede (4.1%), Whole milk yogurt, fruit (1.3%), Mousse (1%), Garlic (0.8%) |
| Morin | 0.0 (0.1) | 0.0 (0.0) | 0.0 | Strawberries (75.9%), Whole milk yogurt, fruit (13.5%), Mousse (10.0%) |
| Isoflavonoids | 2.1 (7.6) | 0.1 (1.4) | 0.1 | Tofu (44.9%), Soya milk (26.0%), Beansprouts (23.3%), Vegeburger (2.5%), Soya mince (1.3%), White bread (0.8%) |
| Daidzein | 0.7 (2.6) | 0.0 (0.4) | 0.0 | Tofu (48.9%), Soya milk (23.8%), Beansprouts (19.8%), Vegeburger (3.4%), Soya mince (1.8%), Doughnuts, jam (0.8%), White bread (0.8%) |
| Genistein | 1.3 (4.6) | 0.0 (0.9) | 0.1 | Tofu (40.6%), Soya milk (29.0%), Beansprouts (25.6%), Vegeburger (2%), Soya mince (1.1%), White bread (0.9%) |
| **Lignans** | 2.7 (1.4) | 2.5 (1.5) | 0.1 | Old potatoes (45.3%), Broccoli (7.7%), Black tea (7.5%), Potatoes (6.9%), Chips, straight cut (3.4%), Vegetable soup (2.3%), Red wine (2%), Oranges (1.9%), Muesli (1.6%), Cabbage (1.3%) |
| Secoisolariciresinol | 0.4 (0.2) | 0.4 (0.2) | 0.0 | Black tea (38.3%), Old potatoes (9.4%), Coffee (5.8%), Carrots (5.8%), Red wine (5.3%), Brazil nuts (4.1%), Kiwi fruit (2.7%), Broccoli (2.2%), Apricots (2.1%) |
| **Other (poly)phenols** | 34.1 (30.8) | 24.1 (30.2) | 1.6 | Bran Flakes (21.3%), Coffee (21%), All-Bran (17.7%), Wholemeal bread (12.3%), Spaghetti (6.2%), Brown bread (4.0%), Red wine (3.6%), Muesli (3.4%), Shredded Wheat (2.2%) |
| Tyrosols | 1.5 (2.5) | 0.7 (1.9) | 0.1 | Red wine (66.7%), White wine (9.7%), Olive oil (9.5%), Fat spread (7.2%), Lager (2.9%), Sherry (2.9%) |
| Vanillin | 0.0 (0.0) | 0.0 (0.0) | 0.0 | Lager (39.6%), Sherry (30.3%), Spirits (18.4%), Vermouth (7.6%), Trifle (4.1%) |
| Furanocoumarins | 0.2 (0.2) | 0.2 (0.2) | 0.0 | Celery (98.2%), Lasagne (0.9%), Moussaka (0.9%) |
| Hydroxycoumarins | 0.1 (0.3) | 0.0 (0.2) | 0.0 | White wine (84.7%), Lager (14.2%) |
| Alkylmethoxyphenols | 3.3 (3.1) | 2.1 (3.9) | 0.2 | Coffee (91.7%), Fat spread (7.5%), Lager (0.7%) |
| Alkylphenols | 24.9 (29.6) | 12.8 (27) | 1.2 | Bran Flakes (29.2%), All-Bran (24.2%), Wholemeal bread (16.8%), Spaghetti (8.5%), Brown bread (5.5%), Muesli (4.7%), Shredded Wheat (3%), Coffee (2.9%), Digestive biscuits, chocolate (2.0%) |
| **Phenolic acids** | 1076.9 (839.9) | 782.7 (1132.1) | 50.4 | Coffee (80.5%), Black tea (8.5%), Cashew nuts (1.2%), Old potatoes (1.1%), Apples (0.9%), Raspberries (0.7%), Carrots (0.8%), Brown rice (0.6%) |
| Hydroxybenzoic acids | 113.9 (68) | 105.6 (101.6) | 5.3 | Black tea (69.1%), Cashew nuts (11.4%), Raspberries (6.5%), Strawberries (2%), Red wine (1.7%), Carrots (1.5%), Garlic (1.3%), Cauliflower (1.1%), Walnuts (0.8%) |
| Benzoic acid | 0.0 (0.0) | 0.0 (0.0) | 0.0 | Drinking chocolate powder (97.0%), Fruitcake (3.0%) |
| Gallic acid | 29.7 (21.1) | 26.8 (28.7) | 1.4 | Black tea (75.8%), Cashew nuts (17.3%), Red wine (3.4%), Bananas (1.6%), Mushrooms (0.4%), Cauliflower (0.4%), Apple juice (0.4%) |
| Vanillic acid | 0.8 (0.4) | 0.8 (0.5) | 0.0 | Carrots (28.0%), Coffee (11.9%), Red wine (11.2%), White rice (11.0%), Fruit 'n Fibre (7.2%), Vegetable soup (3.4%), Brown rice (3.3%), Raspberries (3.3%), Lager (2.5%) |
| Syringic acid | 1.2 (1.6) | 0.6 (0.8) | 0.1 | Walnuts (63.1%), Cauliflower (17%), Red wine (6.4%), Apricots (2.2%), Apple chutney (1.5%), Fat spread (0.9%), Drinking chocolate powder (0.7%), Brown bread (0.7%), Rose wine (0.6%), Semi-sweet biscuits (0.5%) |
| Ellagic acid | 12.2 (20.6) | 7.2 (10.5) | 0.6 | Cashew nuts (64.3%), Strawberries (15.4%), Raspberries (13.8%), Whole milk yogurt, fruit (2.8%), Mousse (2.0%), Walnuts (1.1%) |
| Ellagitannins | 5.5 (7.2) | 4.6 (7.0) | 0.3 | Raspberries (99.3%), Ice cream, non-dairy, vanilla (0.7%) |
| Hydroxycinamic acids | 962.9 (854.2) | 639.3 (1173.7) | 45.1 | Coffee (90.0%), Black tea (1.3%), Old potatoes (1.2%), Apples (1%), Brown rice (0.7%), Sunflower seeds (0.4%), Prunes (0.4%), Carrots (0.4%), White rice (0.4%), Red wine (0.4%) |
| Caffeic acid | 251.0 (238.7) | 156.9 (335.4) | 11.7 | Coffee (96.2%), Carrots, old (0.8%), Carrots, young (0.7%), Broccoli (0.5%), Tomatoes (0.3%), Old potatoes (0.3%), Apples (0.3%), Vegetable soup (0.2%), Red wine (0.2%), Plums (0.1%) |
| Ferulic acid | 53.6 (37.5) | 47.2 (47.9) | 2.5 | Coffee (64.8%), Brown rice (11.8%), White rice (6.4%), Fruit 'n Fibre (3.7%), Brown bread (2.7%), Rice Krispies (2.6%), Orange juice (1.3%), Digestive biscuits, chocolate (1.1%), Spinach (0.7%), Dark chocolates (0.6%) |
| Sinapic acid | 1.9 (1.5) | 1.5 (1.8) | 0.1 | Cauliflower (38.4%), Fruit 'n Fibre (23.4%), Broccoli (14.5%), Orange juice (14.3%), Lager (3.1%), Digestive biscuits, chocolate (1.8%), Grapefruit (1%), Red wine (1.0%) |
| Cinamic acid | 0.2 (0.2) | 0.1 (0.2) | 0.0 | Orange juice (59.6%), Strawberries (23.3%), Coffee (7.0%), Whole milk yogurt, fruit (4.2%), Mousse (3.1%), Raspberries (2.6%) |
| Hydroxyphenylacetic acids | 0.1 (0.2) | 0.0 (0.1) | 0.0 | Red wine (47.7%), White wine (35.5%), Lager (16.4%) |
| **Stilbenes** | 1.0 (1.9) | 0.3 (1.5) | 0.0 | Red wine (77.8%), White wine (16.6%), Grapes (2.6%), Strawberries (1.8%) |
| Resveratrol | 0.3 (0.5) | 0.1 (0.3) | 0.0 | Red wine (59.4%), White wine (22.2%), Grapes (8.7%), Strawberries (6.0%), Whole milk yogurt, fruit (1.1%), Rose wine (0.9%), Mousse (0.8%) |

EPIC-Norfolk FFQ: European Prospective Investigation into Cancer and Nutrition food frequency questionnaire

**Table S4 Association between PPS-M and cardiometabolic health measurements**

| Cardiometabolic health measurements | Standard β (95%CI) | *P* value |
| --- | --- | --- |
| ASCVD risk score | -0.02 (-0.06, 0.02) | 0.50 |
| HeartScore | -0.01 (-0.06, 0.03) | 0.42 |
| SBP | -0.01 (-0.15, 0.12) | 0.85 |
| DBP | -0.07 (-0.20, 0.07) | 0.34 |
| HDL-C | 0.06 (-0.06, 0.18) | 0.30 |

PPS-M, metabolic signature of (poly)phenol-rich dietary score; SBP, systolic blood pressure; DBP, diastolic blood pressure; HDL-C, high-density lipoproteins cholesterol; ASCVD risk score, atherosclerotic cardiovascular disease risk score
